# Autotoxin affects the rhizosphere microbial community structure by influencing the secretory characteristics of grapevine roots

**DOI:** 10.3389/fmicb.2022.953424

**Published:** 2022-07-26

**Authors:** Qianwen Liu, Liheng Zhang, Lu Wang, Qingchun Wu, Kun Li, Xiuwu Guo

**Affiliations:** ^1^Department of Pomology, College of Horticulture, Shenyang Agricultural University, Shenyang, China; ^2^Dalian Academy of Agricultural Sciences, Dalian, China

**Keywords:** ^13^CO_2_ labeling, DNA stable isotope probing, autotoxin, root exudates, replanting disease

## Abstract

Autotoxins secreted by roots into the soil can trigger rhizosphere microecological imbalances and affect root secretory properties resulting in conditions such as replanting disease. However, information on the effect of autotoxins on root secretion characteristics and regulation of the composition of rhizosphere microorganisms by altered root exudates is limited. In this study, autotoxin ρ-hydroxybenzoic acid (4-HBA) was added to the soil of potted grapevine seedlings, CO_2_ pulse-labeling, and DNA stable isotope probing were used to track the rhizosphere microbiome that assimilates root exudates. Bacterial and fungal microbiomes that assimilated plant-derived carbon were identified by high-throughput sequencing. Results showed that 4-HBA treatment altered bacterial and fungal communities in ^13^C-labeled organisms, with a lower abundance of beneficial bacteria (e.g., *Gemmatimonas*, *Streptomyces*, and *Bacillus*) and a higher abundance of potential pathogen fungi (e.g., *Fusarium*, *Neocosmospora*, *Gibberella*, and *Fusicolla*) by changing the composition of root exudates. The exogenous addition of upregulated compound mixtures of root exudates reduced the abundance of beneficial bacterial *Bacillus* and increased the abundance of potential pathogen fungi *Gibberella*. These results suggest that 4-HBA can alter root secretion properties and altered root exudates may enrich certain potential pathogens and reduce certain beneficial bacteria, thereby unbalancing the structure of the rhizosphere microbial community.

## Introduction

The rhizosphere is the narrow soil zone influenced by root secretions, containing up to 10^11^ microbial cells per gram root (Egamberdieva et al., 2008). This highly complex and variable plant-associated microbial community, referred to as the plant’s second genome (Berendsen et al., 2012), critically impacts plant growth and development. The rhizosphere microbial community is affected by various factors such as plant genotype, species, soil physicochemical properties, and cropping regime (Bakker et al., 2012). Plant-rhizosphere microorganism interaction can be beneficial, including host symbiosis, or detrimental, including pathogens and predator, and occasionally even neutral (Bever et al., 2012). Replanting disease is a typical example of negative interactions between plants and rhizosphere microbes. Owing to increasing land scarcity and agro-industrialization, many crops are planted on the same land for long durations, resulting in replanting disease. This phenomenon not only exists in fruit trees (Waschkies et al., 1994; Benizri et al., 2005; Weiß et al., 2017; Huang et al., 2018) but also commonly occurs in field crops (Li et al., 2019), vegetables (Lee et al., 2021), flowers (Lu et al., 2021), and medicinal plants (Ren et al., 2017). Therefore, it is necessary to provide insights into the mechanism of replanting disease, its solution, and the establishment of sustainable agroecosystems.

Replanting disease is a complex phenomenon, and influenced by multiple factors, including nutritional imbalance (Rao et al., 2021), the autotoxicity of root exudates (Chi et al., 2013; Zhang et al., 2020) and the shifts in the microbial community (Zhu et al., 2018; Chen et al., 2020a; Liu et al., 2021). Soil microflora disorder has been regarded as one of the critical causes for the continuous cropping obstacles (Li et al., 2022), and autotoxin played vital roles in altering the bacterial and fungal composition in replanted soil (He et al., 2021). Autotoxins from root exudates of different plants have been identified, including phenolic acids, organic acids, alkaloids, fatty acids, terpenoids, flavonoids, and saponins (Ambika, 2013). Hofmann et al. (2009) reported that after long-term continuous cropping, apple roots will secrete a large amount of flavonoids—phloridzin to attract specific pathogenic microorganisms, which may be an important cause of apple replanting diseases. The accumulated autotoxins in rhizosphere soil of consecutively monocultured *P. heterophylla*, increased the harmful microorganisms and decreased beneficial microorganisms, resulting in an imbalance of microbial community structure and the degradation of soil ecological function (Lin et al., 2015). Exogenous addition of phenolic acids changed the soil microbial community structure and inhibit the growth of peanut plants (Li et al., 2014a). Disruption of the ratio of beneficial and pathogenic microorganisms in the rhizosphere soil and the accumulation of pathogenic microorganisms after long-term monoculture of plants are the major obstacles to continuous cropping (Li et al., 2014b; Huang et al., 2018), and autotoxins play an important role in this process.

Grapevines have a long history of cultivation in China. However, the occurrence of grapevine replanting disease in old vineyards and nurseries has become a common production problem worldwide (Waschkies et al., 1994; Westphal et al., 2002; Manici et al., 2017; Liu et al., 2021). Our previous studies have shown that the rhizosphere soil microbial community changed significantly after long-term grapevine continuous cropping (Liu et al., 2021) and identified autotoxins such as ρ-hydroxybenzoic acid (4-HBA) from grapevine root exudates and rhizosphere soil (Guo et al., 2010; Li et al., 2011). Exogenous 4-HBA is quickly adsorbed by soil, transformed by soil microorganisms (Wang et al., 2017a), changes the soil microbiome diversity (Wang et al., 2019a), and alter the root secretion characteristics (Liu et al., 2019). Root exudates serves as an energy and nutrient resource for microorganisms and plays an important role in regulating the dynamics of microbial populations (Bais et al., 2006; Olanrewaju et al., 2019). Therefore, we hypothesized that autotoxins could affect the characteristics of root exudates and that the altered secretory compounds recruited certain microorganisms and altered the rhizosphere microbial community. Recent studies have mostly addressed the direct effects of autotoxins on rhizosphere microbial communities (Zhang et al., 2016; Zhou and Wu, 2018). However, limited information is available regarding the effect of autotoxins on root secretion characteristics and how changes in root exudates affect the composition and function of rhizosphere microorganisms.

In this study, we used a CO_2_ pulse-labeling and DNA stable isotope probing (DNA-SIP) technique to track the rhizosphere microbiome that assimilates root exudates. The community diversity and composition of bacteria and fungi that assimilate root exudates in rhizosphere soil were characterized by high-throughput sequencing. The aims of this study were to (i) explore the effect of exogenous autotoxin on grapevine root exudates using metabolomics technology, (ii) identify and compare the changes in bacterial and fungal communities assimilating root exudates under exogenous autotoxin by DNA-SIP combined with high-throughput sequencing technology, and (iii) identify compounds that were differently exuded by grapevine roots after autotoxin treatment, and add them back into the soil to examine whether these compounds affect the microbiome.

## Materials and methods

### Plant cultivation and treatments

Cutting seedlings of the grapevine rootstock “Beta” (*Vitis riparia* × *Vitis labrusc*a) were planted in a pot (18 cm × 14 cm × 12 cm, 2.5 L) with 1.8 Kg soil (dry soil) and grown in a greenhouse with an average day/night temperature of 26/18°C. When seedlings developed 4 true leaves, 30 pots of seedlings were poured with 4-HBA solution to make its final concentration in the soil is 1 mg.g^–1^ (soil, T), the other 30 pots were poured with equal volume of distilled water (CK). Soil moisture was maintained at 40–60% of water-holding capacity. The ^13^CO_2_ pulse-labeling was performed 20 days later. Plant height was measured every 5 days during the period.

### ^13^CO_2_-labeling and sampling

**FIGURE 1:**
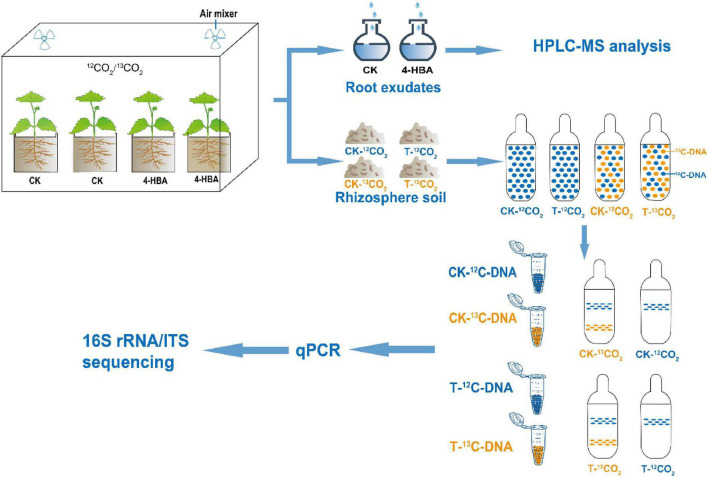
Schematic representation of the experimental approach used for identifying the active microbiome associated with rhizosphere soil of grapevines.

^13^CO_2_ pulse-labeling was carried out in two climate chambers and labeled with ^13^CO_2_ from 8 a.m. to 2 p.m. (6 h) for seven consecutive days (Figure 1). Detailed labeling process is described in Supplementary Materials and Methods. Three days after labeling, rhizosphere soils were separately sampled from 15 seedlings of 4 treatments (CK-^13^CO_2_/T-^13^CO_2_/CK-^12^CO_2_/T-^12^CO_2_), this experiment was performed on three biological replicates, with five seedlings per biological replicate; and seedlings after collecting rhizosphere soil are used to collect root exudates, this experiment was performed on six biological replicates, with five seedlings per biological replicate. The dry and fresh weights of the shoots and roots of the seedlings after collecting root exudates were determined. ^13^C abundance of rhizosphere soil was determined by isotope ratio mass spectrometry (Isoprime100, IRMS, United Kingdom) coupled to an elemental analyzer (Elementar vario PYRO cube, EA, Germany).

### Soil DNA extraction, gradient fractionation and quantitative real-time PCR

The total soil genomic DNA was extracted from 0.3 g of soil using a Power Soil DNA Isolation Kit (MOBIO Laboratories Inc., Carlsbad, CA, United States) according to the manufacturer’s instructions. ^13^C-enriched (“heavy”) DNA was separated from non-labeled (“light”) DNA by CsCl equilibrium density-gradient centrifugation (Neufeld et al., 2007). Briefly, 3.0 ng of rhizosphere soil DNA was mixed well in 5.1 mL Quick-Seal polyallomer tubes (13 × 51mm, Beckman Coulter, Pasadena, CA, United States) with CsCl stock solution and gradient buffer to a final buoyant density (BD) of 1.725 g.mL^–1^. Ultracentrifugation was performed in a vertical rotor (VTi 65.2, Beckman Coulter Inc., Palo Alto, CA, United States) at 45 000 rpm (190,000 × *g*) 44 h at 20°C. DNA fraction was carried out by displacing the gradient medium with sterile water from the top of the ultracentrifuge tube using a NE-1000 single syringe pump (New Era Pump Systems Inc., Farmingdale, NY, United States) with a precisely controlled flow rate of 0.38 mL.min^–1^. Fifteen DNA gradient fractions were generated with equal volume of about 380 μL. The BD of each fraction was determined using an AR200 digital hand-held refractometer (Reichert Inc., Buffalo, NY, United States). Nucleic acids were separated from CsCl solution by precipitation in 550 μL of PEG 6000 solution (30% PEG 6000, 1.6 M NaCl) at 37°C for 1 h, followed by centrifugation at 13 000 × *g* for 30 min. The fractionated DNA was washed twice with 70% ethanol and dissolved in 30 μl of TE buffer (Jia and Conrad, 2009).

Quantitative real-time PCR was used to determine abundance of 16S rRNA gene copy numbers. The PCR reactions were conducted using an ABI Real-Time 7500 system (Applied Biosystems, Waltham, MA, United States). The primer pairs were summarized in Supplementary Table 1. Triplicate assays were conducted for each sample, detailed processes are provided in Supplementary Materials and Methods.

### Microbial diversity analysis

The hypervariable V4-V5 region of bacterial 16S rRNA gene fragments was amplified using the 515F/907R primer set. Partial ITS amplicons were produced using the ITS1F/ITS2R primer set. Finally, purified amplicons were sequenced on the Illumina MiSeq PE300 platform (Illumina, San Diego, CA, United States) according to standard protocols of Majorbio Bio-Pharm Technology Co., Ltd. (Shanghai, China). Raw reads were deposited into the NCBI SRA database under accession number PRJAN826998. Detailed processes of sequencing and data analysis is described in Supplementary Materials and Methods.

### Root exudate collection and ultra high performance liquid chromatography-mass spectrometry analysis

The plant roots were washed with distilled water and then transferred to a container wrapped in aluminum foil containing 1 L of distilled water to collect the root exudates. During the period, the water was aerated for 15 min at 45-min intervals using an air pump. After 72 h of collection, the collected root exudates were freeze-dried and were dissolved with 100 μL of acetonitrile-ultrapure water solution (1:1, v/v), centrifuged at 14,000 × *g* at 4°C for 15 min, and the supernatant were analyzed using an ultra high performance liquid chromatography-mass spectrometry (UHPLC-MS) system, consisting of Agilent 1290 Infinity LC UHPLC (Agilent Technologies, Inc., Santa Clara, CA) coupled to a Triple TOF 5,600 + High Resolution Mass Spectrometer (AB Sciex, Framingham, MA, United States). The UHPLC separation was carried out on a Waters ACQUITY UPLC BEH Amide column (1.7μm, 2.1 mm × 100 mm). The detailed instrument parameters and data analysis procedures are described in Supplementary Materials and Methods.

### Impacts of differential root exudate on soil microbiome community

To examine the effect of differential root exudates of grapevine after 4-HBA treatment on soil microbiome community, we selected significantly up- and down-regulated representative compounds from the four categories of sugars, amino acids, organic acids, and secondary metabolites, and formulated them into mixed water solutions (Supplementary Table 2).

Fifteen grams of soil was placed into each flask. Flasks were pre-incubated in a growth chamber at 30°C for 1 week to allow the soil microbiome to acclimatize. Then add 1.5 mL of exudate compound solution to each flask for a total of 12 additions for 45 days in a growth chamber at 30°C. We applied three treatments: (1) Up, up-regulated mixture solution; (2) Down, down-regulated mixture solution; and (3) control, sterilized distilled water. Each treatment consisted of 15 replicates. After 45 d, within each treatment, samples from every 5 randomly were pooled, thereby yielding 3 biological replicates per treatment. All samples were immediately stored at −80°C prior to subsequent soil fungal and bacterial community analysis.

### Statistical analyses

The δ^13^C value of four treatments (CK-^13^CO_2_/T-^13^CO_2_/CK-^12^CO_2_/T-^12^CO_2_), the alpha diversity index, relative abundance of bacteria and fungi at the phylum or genus level between four treatments (CK-^12^C, CK-^13^C, T-^12^C, T-^13^C) were evaluated by one-way analysis of variance (ANOVA) and Turkey’s test. Student’s *t*-test was used to test the difference of alpha diversity index, relative abundance of bacteria and fungi at the phylum or genus level between CK-^12^C and CK-^13^C or between T-^12^C and T-^13^C or between CK-^13^C and T-^13^C, and the peak areas of each categorical metabolite in the root secretions of CK and 4-HBA treatments. The relative abundance of bacteria and fungi at genus level among up- or down-regulated compounds mixtures addition were analyzed by one-way ANOVA and Turkey’s test. The one-way ANOVA and Student’s *t*-test were performed using SPSS 19.0 Statistical software (SPSS, Chicago, IL, United States), and *p* < 0.05 was considered significant.

## Results

### Plant performance and ^13^C enrichment in rhizosphere soil

**FIGURE 2:**
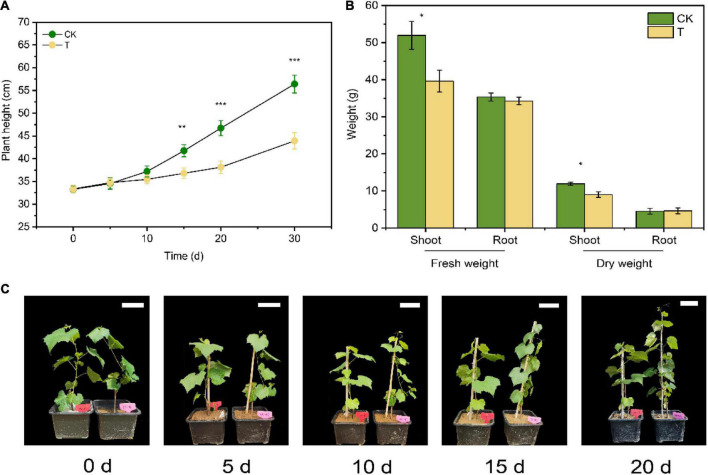
Effects of 4-HBA treatment (T) on seedlings growth. **(A)** Effects of 4-HBA treatment (T) on plant height of grapevine seedlings. **(B)** Dry and fresh weights of shoots and roots of seedlings treated with 4-HBA (T) for 30 days. **(C)** Pictures of seedlings growth during 4-HBA treatment. The white scale bar in the picture represents 10 cm. The asterisk indicates significant differences between CK and 4-HBA treatment (T) (Student’s *t*-test, *p < 0.05; **p < 0.01; ***p < 0.001).

After 4-HBA treatment for 10d, the plant height of grapevine seedlings was inhibited, but there was no significant difference with CK. After 4-HBA treatment for 20d, there was a significant difference (*p* < 0.001) in seedlings height between 4-HBA and CK (Figures 2A,C). When the treatment reached 30d, the dry weight and fresh weight of shoots decreased by 24.4 and 23.7%, respectively, compared with CK. While root biomass was not significantly changed after 4-HBA treatment (Figure 2B). The overall isotopic signature of δ^13^C demonstrated that the rhizosphere soil was significantly (*p* < 0.05) enriched in ^13^C under ^13^CO_2_ conditions (Supplementary Figure 1), indicating the successful incorporation of grapevine ^13^C-rhizodeposits into the rhizosphere soil. Furthermore, the δ^13^C value was significantly higher following the 4-HBA treatment than in CK under ^13^CO_2_ conditions, indicating that 4-HBA treatment promoted the secretion of more root exudates into the rhizosphere soil.

### ^13^C enrichment and distributions of nucleic acids in centrifugation gradients

**FIGURE 3:**
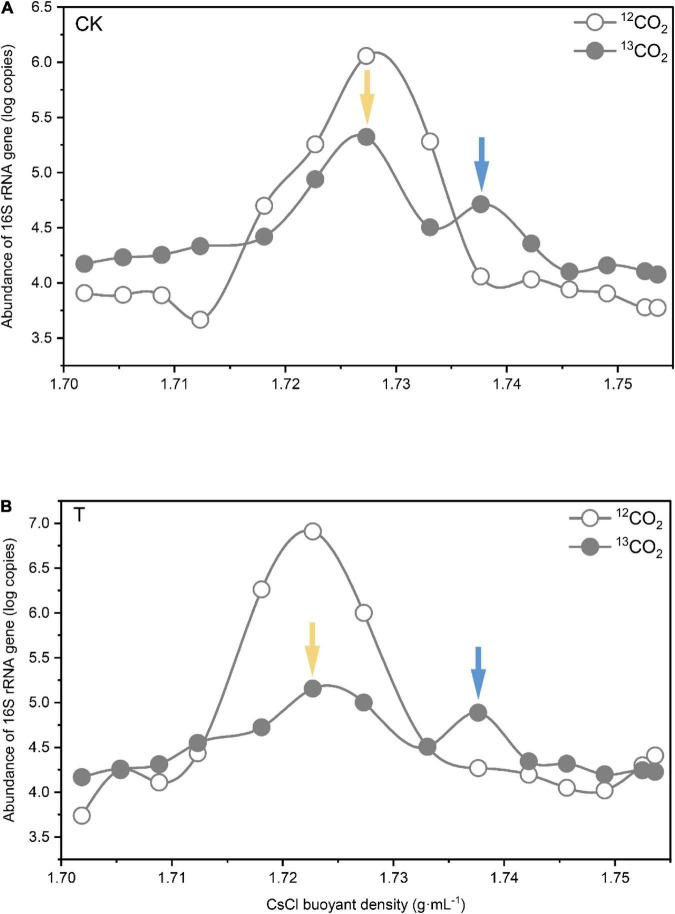
Quantitative distribution of bacterial 16S rRNA gene copy numbers across the entire buoyant density gradient of the fractionated DNA from the rhizosphere soil incubated with ^13^CO_2_ or ^12^CO_2_ after CK **(A)** and 4-HBA treatment **(B)**. The values given are the means of three separate treatments. The blue and yellow arrows indicate the locations of ^13^C-and ^12^C-labeled DNA, respectively, which were used to construct amplicon libraries for high-throughput pyrosequencing.

To identify microorganisms assimilating ^13^C-labeled plant-derived carbon, DNA was isopycnically centrifuged and fractionated based on buoyant density. Quantification of 16S rRNA genes in the gradient fraction by real-time PCR revealed similar abundance patterns among the gradient fractions of CK and 4-HBA treatment samples (Figures 3A,B). The 16S rRNA gene abundance in ^12^CO_2_ microcosms showed a unique peak in the light fraction around a BD of 1.725–1.730 g.mL^–1^, whereas we observed two peaks of 16S rRNA gene abundance in the ^13^CO_2_ microcosms, one in the heavy fraction (1.735–1.740 g.mL^–1^) and the other in the light fraction (1.725–1.730 g.mL^–1^). These results indicate that ^13^C-labeled plant-derived carbon enters the rhizosphere and is successfully assimilated by the microorganisms. Based on these results, fractions with a BD of approximately 1.725–1.730 g.mL^–1^ CsCl in the light fraction and 1.735–1.740 g.mL^–1^ CsCl in the heavy fraction were considered ^12^C-labeled and ^13^C-labeled DNA, respectively. To investigate the effects of root exudates affected by 4-HBA on rhizosphere bacteria and fungi, ^12^C-labeled and ^13^C-labeled DNA in ^13^C microcosms under CK and 4-HBA treatments were further sequenced by high-throughput sequencing.

### Diversity and composition of ^12^C- and ^13^C-labeled bacteria

In total, 672 821 quality-filtered paired-end 16S rRNA sequences were obtained from 12 samples, ranging from 48,338 to 67,275 sequences per sample. Bacterial richness indices (Ace and Chao) were significantly reduced in the ^13^C-labeled fraction compared with the ^12^C-labeled fraction in the CK and 4-HBA treatments. Bacterial diversity indices (Shannon and Simpson) indicated that Shannon’s index of T-^13^C was significantly lower than that of the other three treatments, and there was no significant difference in Simpson’s index (Table 1).

**TABLE 1:** Alpha diversity indices of bacterial and fungal communities in soil samples of ^12^C- and ^13^C-labeled DNA under CK and 4-HBA treatments (T).

	Treatment	Ace	Chao	Shannon	Simpson
Bacteria	CK-^12^C	2,938 ± 42 a***	2,897 ± 36 a***	6.36 ± 0.04 a	0.0072 ± 0.0014a
	CK-^13^C	2,483 ± 8b	2,469 ± 17 b	6.18 ± 0.003 a	0.0046 ± 4.11E-04 a
	T-12C	2,815 ± 22 a***	2,786 ± 19 a***	6.23 ± 0.009 a***	0.0059 ± 1.48E-04 a
	T-^13^C	2,365 ± 30b	2,386 ± 24 b	6.06 ± 0.009 b	0.006 ± 1.81E-04 a
Fungi	CK-^12^C	858 ± 21 a ***	861 ± 19 a***	3.89 ± 0.025 b	0.068 ± 0.002 b***
	CK-^13^C	557 ± 21 c	559 ± 22 c	4.36 ± 0.014 a***	0.0406 ± 6.75E-04 c
	T-^12^C	705 ± 21 b***	704 ± 18 b***	3.419 ± 0.044 c	0.096 ± 0.008 a***
	T-^13^C	405 ± 7 d	416 ± 12 d	3.4 ± 0.015 c	0.076 ± 1.98E-04b

Values were mean ± standard error (n = 3). Different letters indicated significant differences among the four treatments at the p < 0.05 (one-way ANOVA, Trukey’s test). ***Indicated significant difference at p < 0.001 between CK-^12^C and CK-^13^C or between T-^12^C and T-^13^C (Student’s t-test).

**FIGURE 4:**
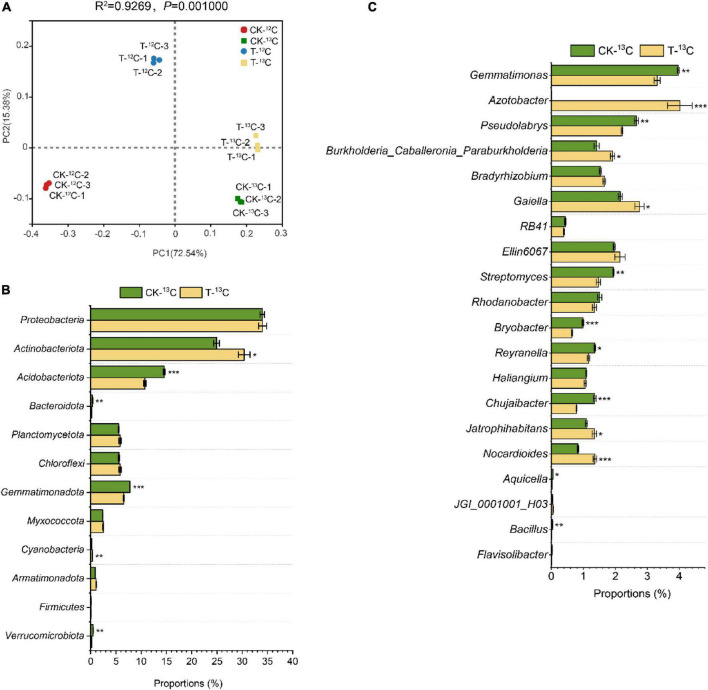
**(A)** Principal co-ordinates analysis based on Bray–Curtis distances of bacterial communities in soil samples of ^12^C and ^13^C-labeled DNA under CK and 4-HBA treatments (T) (ADONIS test). **(B)** Relative abundance of different bacterial taxa at the phyla level and major variations in soil samples between CK-^13^C and T-^13^C. **(C)** Relative abundance of different bacterial taxa at the genus level and major variations in soil samples between CK-^13^C and T-^13^C. Bars represent means ± standard errors (*n* = 3). The asterisk indicates significant differences between CK-^13^C and T-^13^C (Student’s *t*-test, *p < 0.05; **p < 0.01; ***p < 0.001).

Principal coordinate analysis (PCoA) with the Bray-Curtis distance showed that the contribution of the first two axes to the bacterial communities was 72.54 and 15.38%. Each treatment formed evident separate groups, indicating that the bacterial communities were affected by root exudates and 4-HBA treatment. Furthermore, the bacterial communities of the ^12^C-labeled fraction distinctly separated from those of the ^13^C-labeled fraction in the first axis, and its contribution is as high as 72.54% (Figure 4A). However, those of CK and 4-HBA treatments were separated along the second axis, indicating that root exudates were responsible for more variation in the bacterial community than the 4-HBA treatment.

There were substantial differences in the bacterial community structures between the ^13^C- and ^12^C-labeled fractions of CK and 4-HBA treatments. At the phylum level, the relative abundance of *Actinobacteriota*, *Gemmatimonadota*, and *Myxococcota* was significantly higher in the ^13^C-labeled fraction than in the ^12^C-labeled fraction for CK and 4-HBA treatments, whereas the relative abundance of *Acidobacteriota*, *Bacteroidota*, *Cyanobacteria*, and *Firmicutes* showed the opposite trend. In addition, *Armatimonadota* was more abundant in the 4-HBA treatment than in the CK treatment in the ^12^C- and ^13^C-labeled fractions (Supplementary Figures 2A and Supplementary Table 3). The relative abundances of *Gemmatimonas, Gaiella, Ellin6067, Streptomyces, Rhodanobacter, Reyranella, Haliangium, Chujaibacter, Jatrophihabitans*, and *Nocardioides* at the genus level were significantly higher in the ^13^C-labeled fraction than in the ^12^C-labeled fraction in CK and 4-HBA treatments. In contrast, *RB41*, *Bryobacter*, *Aquicella*, *JGI_0001001_H03*, *Bacillus*, and *Flavisolibacter* were found in significantly lower proportions in the ^13^C-labeled fraction than in the ^12^C-labeled fraction. The relative abundance of *Azotobacter*, *Jatrophihabitans*, and *Nocardioides* were significantly increased in 4-HBA treatment compared to CK in ^12^C-and ^13^C-labeled fractions (Supplementary Figure 2B and Supplementary Table 4).

To investigate the effect of root exudates under 4-HBA treatment on the rhizosphere bacterial community, we further analyzed the differences between the bacterial communities of CK-^13^C and T-^13^C. The relative abundance of *Actinobacteriota* (logFC, *p*: 0.09, 1.26E-02) and *Cyanobacteria* (0.23, 7.76E-04) significantly increased in T-^13^C compared with CK-^13^C samples by 22.00 and 75.00%, whereas the abundances of *Acidobacteriota* (−0.13, 2.51E-04), *Bacteroidota* (−0.19, 9.70E-03), *Gemmatimonadota* (−0.07, 4.84E-04), and *Verrucomicrobiota* (−0.38, 2.94E-03) were reduced by 26.25, 34.21, 15.50, and 58.00% (Figure 4B and Supplementary Table 5). At the genus level, the relative abundances of *Azotobacter* (3.16, 4.77E-04), *Burkholderia-Caballeronia-Paraburkholderia* (0.13, 1.04E-02), *Gaiella* (0.11, 1.78E-02), *Jatrophihabitans* (0.09, 3.24E-02), and *Nocardioides* (0.21, 7.51E-04) were significantly higher in T-^13^C than that in CK-^13^C by 965.35%, 34.51, 27.91, 21.82, and 63.86%, respectively. While the relative abundance of *Gemmatimonas* (−0.08, 2.40E-03), *Pseudolabrys* (−0.08, 3.11E-03), *Streptomyces* (−0.12, 2.99E-03), *Bryobacter* (−0.18, 6.90E-04), *Reyranella* (−0.07, 1.19E-02), *Chujaibacter* (−0.24, 2.00E-04), *Aquicella* (−0.29, 2.26E-02), and *Bacillus* (−0.42, 3.48E-03) was significantly reduced by 16.62, 16.98, 24.23, 34.34, 14.71, 42.22, 40.00, and 75.00%, respectively. Notably, the relative abundance of *Azotobacter* increased by 967.7 times (Figure 4C and Supplementary Table 6).

### Diversity and composition of ^12^C- and ^13^C-labeled fungi

We obtained 905 977 high-quality paired-end ITS reads from 12 samples ranging from 64,372 to 82,056 sequences from the 12 samples. The fungal richness indices (Ace and Chao) in the ^13^C-labeled fraction were significantly lower than those in the ^12^C-labeled fraction in CK and 4-HBA treatments. The fungal diversity index (Simpson) showed a similar trend (Table 1).

**FIGURE 5:**
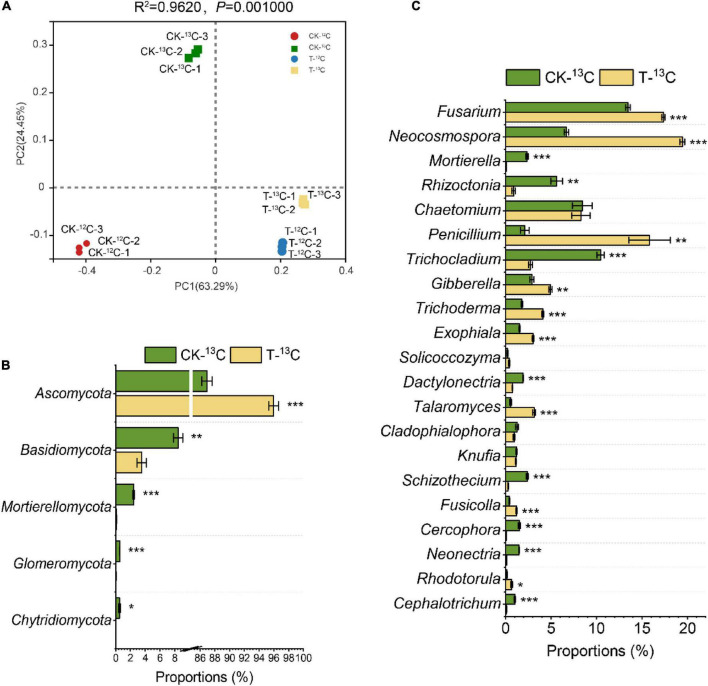
**(A)** Principal co-ordinates analysis based on Bray–Curtis distances of fungal communities in soil samples of ^12^C and ^13^C-labeled DNA under CK and 4-HBA treatments (T) (ADONIS test). **(B)** Relative abundance of different fungal taxa at the phyla level and major variations in soil samples between CK-^13^C and T-^13^C. **(C)** Relative abundance of different fungal taxa at the genus level and major variations in soil samples between CK-^13^C and T-^13^C. Bars represent means ± standard errors (*n* = 3). The asterisk indicates significant differences between CK-^13^C and T-^13^C (Student’s *t*-test, *p < 0.05; **p < 0.01; ***p < 0.001).

PCoA with Bray-Curtis distance showed that the first two axes represented 63.29% (PC1) and 24.45% (PC2) of the variation in fungal communities, respectively. Each sample had distinct groups, indicating that root exudates and 4-HBA treatment significantly affected fungal communities. The PC1 generally distributed the fungal communities along with the CK and 4-HBA treatments, and its contribution was as high as 63.29%, indicating that 4-HBA was responsible for more of the variation in the rhizosphere fungal community than root exudates (Figure 5A).

There were substantial differences in fungal community structures among the four treatments (CK-^12^C, CK-^13^C, T-^12^C, and T-^13^C). At the phylum level, the relative abundance of phylum *Ascomycota* was significantly more in the ^13^C-labeled fraction than the ^12^C-labeled fraction in CK and 4-HBA treatments by 135.07 and 21.74%, respectively. In contrast, *Basidiomycota, Mortierellomycota, Glomeromycota*, and *Chytridiomycota* were significantly less abundant in ^13^C- compared to the ^12^C-labeled fraction (Supplementary Figures 3A and Supplementary Table 7). At the genus level, *Chaetomium*, *Penicillium*, *Trichocladium*, *Trichoderma*, *Exophiala*, *Talaromyces*, *Knufia*, *Schizothecium*, and *Neonectria* were more abundant in the ^13^C- than the ^12^C-labeled fraction, whereas *Mortierella*, *Rhizoctonia*, and *Solicoccozyma* were less abundant in the ^13^C-labeled fraction in the CK and 4-HBA treatments (Supplementary Figure 3B and Supplementary Table 8).

To investigate the effect of root exudates on the fungal community after 4-HBA treatment, we compared the relative abundances of fungi between CK-^13^C and T-^13^C. At the phyla level, except for *Ascomycota* (0.04, 7.78E-04), *Basidiomycota* (−0.38, 5.34E-03), *Mortierellomycota* (−1.41, 3.31E-05), *Glomeromycota* (−1.17, 4.19E-05), and *Chytridiomycota* (−1.39, 1.59E-02) were significantly downregulated (Figure 5B and Supplementary Table 9). At the genus level, *Fusarium* (0.11, 1.79E-04), *Neocosmospora* (0.46, 3.59E-06), *Penicillium* (0.87, 4.17E-03), *Gibberella* (0.23, 1.98E-03), *Trichoderma* (0.35, 7.58E-05), *Exophiala* (0.29, 1.79E-04), *Talaromyces* (0.75, 1.41E-04), *Fusicolla* (0.46, 5.89E-04), and *Rhodotorula* (0.64, 1.24E-02) were more abundant in T-^13^C than CK-^13^C; while *Mortierella* (−1.44, 4.24E-05), *Rhizoctonia* (-0.80, 2.16E-03), *Trichocladium* (−0.58, 6.02E-05), *Dactylonectria* (−0.41, 2.69E-05), *Schizothecium* (−0.95, 4.34E-05), *Cercophora* (−1.25, 3.22E-04), *Neonectria* (−1.09, 1.24E-06), and *Cephalotrichum* (−0.98, 3.95E-04) were significantly less abundant in T-^13^C than CK-^13^C (Figure 5C and Supplementary Table 10).

### Impact of ρ-hydroxybenzoic acid treatment on root exudation profiles

**FIGURE 6:**
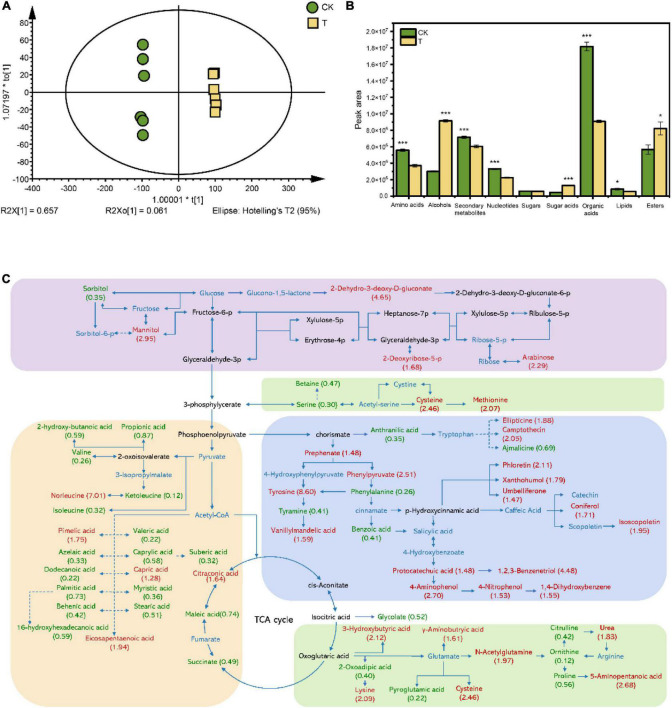
**(A)** Score scatter plot of the OPLS-DA model for identified root exudate differences in the control and 4-HBA treatments. The scatter shape and color represent different experimental groups. CK: Control; T: 4-HBA treatment. **(B)** Peak area of differential metabolite categories. Each bar represents the average of six replicates. The asterisk indicates statistically significant differences between CK and 4-HBA treatment (Student’s *t*-test, *p < 0.05; ***p < 0.001). **(C)** Effects of 4-HBA treatment on the metabolic pathways involving grapevine root exudates. The metabolites in the blue text were detected in the present experiment; the metabolites in red or green text represent significantly upregulated (*p* < 0.05) or downregulated (*p* < 0.05) metabolites compared with CK. The solid and dashed lines represent direct and indirect metabolite pathways, respectively. Numbers in parentheses represent FC values. The figure has been made readable by roughly separating the metabolites into carbohydrate, organic acid, amino acids, and secondary metabolites metabolism, as shown by the purple, yellow, green, and blue rectangle frames.

To establish a mechanistic explanation for the effect of 4-HBA treatment on root secretion and consequently the rhizosphere microbial community, root exudates were collected and analyzed by UHPLC-MS. A total of 708 metabolites were obtained and annotation. The overall exudation patterns from control plants were distinct from those of plants treated with 4-HBA, as demonstrated by their separation in OPLS-DA modeling (Figure 6A). Parameters of variable importance in projection (VIP) score > 1 and *p* < 0.05 were adopted to identify the metabolites responsible for the separation between CK and T. In total, 222 compounds detected in both treatments differed significantly (*p* < 0.05), and the 222 metabolites were identified and divided into broad categories based on their structure (Supplementary Table 11). The sum of the peak areas of differential metabolites in each category indicated that amino acids, secondary metabolites, nucleotides, organic acids, and lipids were significantly reduced after 4-HBA treatment. In contrast, the peak areas of alcohols, sugar acids, and esters were significantly increased (Figure 6B). The number of differentially expressed grapevine metabolites in different categories is shown in Supplementary Figure 4; the number of upregulated amino acids (upregulated: 22; downregulated: 14), secondary metabolites (24; 7), and sugar acids (5; 0) was much higher than that of downregulated metabolites; however, the number of downregulated metabolites was higher in the organic acids (12; 20). The main metabolic pathways of the root exudates detected in this study were established based on the KEGG database (Figure 6C). The KEGG pathways for significant changes are shown in Supplementary Figure 5. Four of the top ten KEGG pathways were related to amino acid synthesis and metabolism, and three were related to organic acid synthesis and metabolism. Overall, 4-HBA treatment affected the secretion of sugars, organic acids, amino acids, and secondary metabolites in grapevine roots.

### Impact of exudation on soil microbial community

**FIGURE 7:**
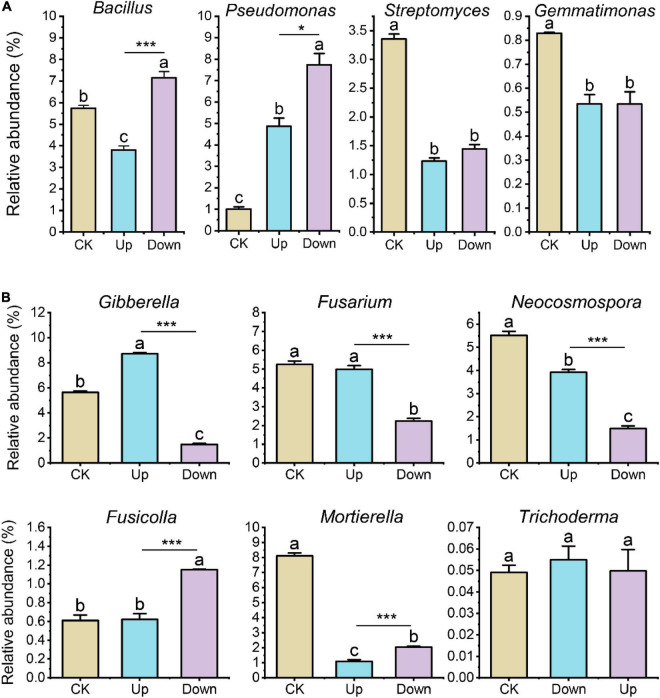
**(A)** Relative abundance of bacterial genera (*Bacillus*, *Pseudomonas*, *Streptomyces*, and *Gemmatimonas*) after adding upregulated or downregulated compound mixtures. **(B)** Relative abundance of fungal genera (*Gibberella*, *Fusarium*, *Neocosmospora*, *Fusicolla*, *Mortierella*, and *Trichoderma*) after adding upregulated or downregulated compound mixtures. Bars represent means ± standard errors (*n* = 3). Different letters indicated significant differences among the three treatments at the *p* < 0.05 (one-way ANOVA, Trukey’s test). The asterisk indicates statistically significant differences between ‘Up’ and ‘Down’ treatment (Student’s t-test, *p < 0.05; ***p < 0.001).

Next, we conditioned the soil by repeatedly adding mixtures of compounds (Supplementary Figure 6) that were either up- or downregulated in the exudates of grapevines after 4-HBA treatment and then investigated the microbial community of the conditioned soil by amplicon sequencing. Soil bacterial and fungal community structure significantly changed after adding upregulated and downregulated mixture solutions (Supplementary Figures 7A,B), especially the relative abundance of *Bacillus* and *Pseudomonas* significantly increased after adding downregulated mixture solutions (Figure 7A). For fungi, the relative abundance of *Gibberella* was significantly increased after adding upregulated mixture solutions compared to other treatments; *Fusarium* and *Neocosmospora* were less abundant, while the relative abundance of *Fusicolla* was significantly increased after adding downregulated mixture solutions compared to other treatments. In addition, *Mortierella* was less abundant after adding differentially regulated compound mixtures, whereas the relative abundance of *Trichoderma* remained unchanged (Figure 7B).

## Discussion

### The bacterial and fungi communities of ^12^C-and ^13^C-fractions are distinct

We found that the structure and diversity of ^13^C-labeled bacterial and fungal communities were significantly different from those of ^12^C-labeled in the control and 4-HBA treatments, indicating that certain bacterial or fungal communities assimilated ^13^C-labeled root exudates. *Proteobacteria*, *Actinobacteriota*, *Acidobacteriota*, and *Bacteroidota*, the dominant bacterial phyla in the rhizosphere soil of grapevines (Supplementary Figure 2A and Supplementary Table 3), are the common inhabitants in the rhizosphere of many plant species (Franke-Whittle et al., 2015; Huang et al., 2018). *Actinobacteriota* was more abundant in ^13^C- than in the ^12^C-labeled fraction (Supplementary Figure 2A and Supplementary Table 3). It is consistent with previous research showing that root exudates of *Arabidopsis thaliana* from the fast plant growth stage enriched the relative abundance of *Actinobacteria* in the soil (Zhao et al., 2021). The relative abundance of some genera of *Actinobacteria* phylum, such as *Gaiella*, *Streptomyces*, *Jatrophihabitans*, and *Nocardioides*, was also higher in the ^13^C-fraction than in the ^12^C-fraction (Supplementary Figure 2B and Supplementary Table 4), suggesting that these bacteria were actively incorporating secreted carbon from grapevine roots. Genus *Gemmatimonas*, a chemoorganotrophic group involved in the decomposition of organic matter and crop residues (Mau et al., 2015; Banerjee et al., 2016), contributes to soil carbon sequestration (Wang et al., 2017b) and exhibited a significantly higher relative abundance in the ^13^C-labeled fraction than in the ^12^C-labeled fraction (Supplementary Figure 2B and Supplementary Table 4), indicating that it could efficiently utilize carbon in root exudates. The relative abundance of the three genera (*Rhodanobacter, Reyranella, Chujaibacter*) belonging to the *Proteobacteria* phylum was also higher in the ^13^C-labeled fraction (Supplementary Figure 2B and Supplementary Table 4), among which *Rhodanobacter* is involved in soil denitrification (Heuvel et al., 2010; Green et al., 2012), a heterotrophic activity, and could use root exudates as a C-source (Guyonnet et al., 2018). Unlabeled microorganisms are considered to degrade unlabeled carbon, such as stable soil organic matter (SOM) (Fontaine et al., 2003; Guyonnet et al., 2018) or possibly from older structural pools (Gkarmiri et al., 2017). The genus *Bryobacter* was more abundant in the ^12^C-labeled fraction (Supplementary Figure 2B and Supplementary Table 4), which was attributed to *Bryobacter* being a slow-growing chemoorganotrophic bacteria (Kulichevskaya et al., 2009), thereby responding slowly to carbon released by the roots. It likely has a role as a diverse degrader of organic matter in tundra soils (Rawat et al., 2012) and is significantly positively correlated with SOM (Li et al., 2021) and soil enzyme activity (Zhou et al., 2019).

*Ascomycota* and *Basidiomycota* were the dominant fungal groups in the grapevine rhizosphere soil. *Ascomycota* is involved in dead plant material and cellulosic plant polymer decomposition (Baldrian et al., 2011). Most *Ascomycota* belong to fast-growing fungal populations (r-strategists) (Bastian et al., 2009; Chen et al., 2015). In this study, *Ascomycota* was more abundant in the ^13^C-labeled fraction than in the ^12^C-labeled fraction (Supplementary Figure 3A and Supplementary Table 7), consistent with a previous study in which members of *Ascomycota* were the first users of freshly photosynthesized carbon (Hannula et al., 2020), and some genera such as *Penicillium* and *Trichocladium* were also more abundant in the ^13^C-labeled fraction (Supplementary Figure 3B and Supplementary Table 8). In contrast, the relative abundance of *Basidiomycota* was lower in the ^13^C-labeled fraction, probably because the members of *Basidiomycota* did not use recently fixed carbon (Hannula et al., 2020). *Mortierella* and *Rhizoctonia* were mainly detected in the ^12^C-labeled fraction (Supplementary Figure 3B and Supplementary Table 8), suggesting that those fungi primarily assimilated unlabeled carbon, possibly from older structural factor pools (Gkarmiri et al., 2017; Wang et al., 2019b).

### Autotoxin treatment changed the bacterial and fungal community in the ^13^C-labeled fraction

The composition of the bacterial and fungal communities actively assimilating root exudates differed between CK and 4-HBA treatments because 4-HBA treatment may change the quality and quantity of root exudates, which can impact soil microorganism abundance and carbon cycling (Haichar et al., 2014). Notably, after 4-HBA treatment, the relative abundance of *Azotobacter* in the ^12^C- and ^13^C-labeled fractions increased by more than 100-fold (Supplementary Figure 2B and Supplementary Table 4). This is consistent with our previous findings that *Azotobacter* abundance in the ^13^C-layer was significantly increased after treating grapevine rhizosphere soil with ^13^C-labeled 4-HBA (Wang et al., 2019a), indicating that *Azotobacter* can utilize 4-HBA in addition to other autotoxins (Wang et al., 2021b). Although the relative abundance of *Actinobacteria* was higher in T-^13^C than in CK-^13^C, the genera *Streptomyces* were less abundant in T-^13^C than in CK-^13^C. *Streptomyces* is the largest genus (more than 500 species) within *Actinobacteria* (Viaene et al., 2016) and produces antibiotics (Toumatia et al., 2015) and synthesizes plant growth hormones such as auxin and cytokinin (Palaniyandi et al., 2013); therefore, it is a novel plant growth-promoting rhizobacterium (PGPR) (Lehr et al., 2007, 2008; Schrey and Tarkka, 2008). Two other genera of potentially beneficial bacteria, *Gemmatimonas* and *Bacillus*, were less abundant in T-^13^C (Figure 4C and Supplementary Figure 6). *Gemmatimonas* occur at a higher frequency in healthy plants than in diseased plants and most likely play a key role in pathogen antagonism and biocontrol programs (Yin et al., 2013). *Bacillus* is a well-known bacterial genus that acts as a PGPR. It can synthesize plant growth hormones to promote plant growth (Amer and Utkhede, 2000), or inhibit pathogens by synthesizing antibiotics (Sivasakthi et al., 2014). In this study, the decrease in the relative abundance of bacterial genera, such as *Gemmatimonas*, *Streptomyces*, and *Bacillus* in the ^13^C-labeled fraction after 4-HBA treatment may be due to the reduced secretion of metabolites that were assimilated by these bacteria, or it may be the result of competition among microorganisms.

Most genera with high abundance in T-^13^C compared to CK-^13^C belonged to the phylum *Ascomycota*, except for the genus *Rhodotorula* (Figure 5C and Supplementary Table 10). Many previous studies have demonstrated that most soil-borne pathogens belong to *Ascomycota* (Ye et al., 2021). *Fusarium* and *Fusicolla* can cause soil-borne diseases in plants (Chen et al., 2020b). *Fusarium* is widespread in soil, and many are pathogenic. For example, *Fusarium solani* and *Fusarium oxysporum* are soil-borne pathogens that cause brown root rot and Fusarium wilt in watermelon, banana, peanut, and tomato plants (Ploetz, 2006; Rojo et al., 2007; McGovern, 2015; Xue et al., 2019). Treatment with autotoxins (e.g., ferulic acid and cinnamic acid) increases *Fusarium* abundance and the incidence of *Fusarium* wilt (Su et al., 2006; Zhou and Wu, 2012), which is consistent with our results. The genus *Neocosmospora*, a potential pathogen, is commonly found in soil and was assigned to the *Fusarium solani* species complex (O’Donnell, 2000; Temporini and VanEtten, 2004; Sandoval-Denis et al., 2018). *Gibberella* is a pathogen (Kebede et al., 2018), and its relative abundance showed significant positive correlations with the contents of individual and total phenolic acids in the rhizosphere soil after continuous cropping of Sanqi ginseng (*Panax notoginseng*) (Wang et al., 2021a) and treatment with autotoxic ginsenosides increased the relative abundance of *Gibberella* (Li et al., 2020). Here, the relative abundance of *Fusarium*, *Fusicolla*, *Neocosmospora*, and *Gibberella* in ^13^C-fraction after 4-HBA treatment was higher than that of CK, and we speculate that this may be due to the altered secretion of some metabolites that are utilized by these fungi after 4-HBA treatment. In addition, the relative abundances of two beneficial fungal genus (*Trichoderma and Mortierella*) also changed. Compared to CK-^13^C, the relative abundance of *Trichoderma* increased significantly in T-^13^C. Gao et al. (2019) reported that the exogenous addition of 4-HBA increased the abundance of *Trichoderma* in the rhizosphere soil of cucumber and speculated that *Trichoderma* might be capable of degrading 4-HBA. Our results indicate that, in addition to the direct effect of 4-HBA, the abundance of *Trichoderma* seem to be influenced by root secretions. In addition, the relative abundance of *Mortierella* decreased significantly in T-^13^C compared to CK-^13^C, which is in accordance with earlier findings of peanut root exudates decreasing the relative abundance of the fungal taxa *Mortierella* (Li et al., 2014a). These results indicate that the abundance of some important fungi and bacteria in the ^13^C-labeled fraction of the rhizosphere soil changed after 4-HBA treatment. We speculate that this change may be due to changes in root secretions after 4-HBA treatment.

### Impact of exudation compounds affected by autotoxin on soil microbial community

To test the above speculation that 4-HBA-affected root exudates affect the abundance of crucial bacteria and fungi, we determined the composition of root exudates after 4-HBA treatment. We found that sugar, amino acids, organic acids (especially fatty acids), and secondary metabolites of roots changed significantly after 4-HBA treatment (Figure 6). These metabolites constitute a significant fraction of exudates, of which sugar is the main carbon source for microbes (Kawasaki et al., 2016). Amino acids are recognized by microbial chemoreceptors that are crucial for the early steps of root colonization (Allard-Massicotte et al., 2016), and organic acids are microbial nutrients (Sasse et al., 2018). To verify whether the changes in rhizosphere microorganisms in the “isotope labeling experiment” were caused by different root exudates, we added mixtures of compounds that were either significantly upregulated or downregulated in the exudates of grapevines after 4-HBA treatment and measured the soil microbial community. The bacterial and fungal community structures in the soil were significantly different after adding different compound mixtures (Supplementary Figures 7A,B). We noticed that the genus *Bacillus* was more abundant after adding the downregulation compound mixture than the other treatments (Figure 7A). This is consistent with our previous “isotope labeling experiment” results that the relative abundance of *Bacillus* was less in the ^13^C-labeled fraction (assimilates root exudates) after 4-HBA treatment (Figure 4C), indicating that some substances that decrease after 4-HBA treatment may be beneficial to *Bacillus* proliferation. We did not focus on the changes in *Pseudomonas* in the “isotope labeling experiment” because of its low abundance. In contrast, the relative abundance of *Pseudomonas* increased after adding upregulation and downregulation compound mixtures compared to CK with the effect of the downregulation compound mixture being more evident, probably because some substances in the upregulation and downregulation compound mixtures favored *Pseudomonas* growth. The upregulation and downregulation compound mixtures contained amino acids and organic acids, and the organic acids in the mixture of downregulated compounds were mainly long-chain organic acids (fatty acids). Recent studies support that the application of long-chain organic acids and amino acids could promote host plant systemic resistance (Yuan et al., 2018) by helping to recruit beneficial microbes such as *Pseudomonas* (Wen et al., 2021). Amino acids are particularly valuable resources for bacteria, and those contained in the mixture of downregulated compounds were phenylalanine, valine, isoleucine, proline, and ornithine, of which *Bacillus subtilis* use proline as a sole carbon or nitrogen source (Moses et al., 2012). The relative abundance of *Streptomyces* and *Gemmatimonas* reduced after adding differentially regulated compound mixtures, indicating that the substances added to the mixture of differentially regulated substances were not the nutrient source for *Streptomyces* and *Gemmatimonas*. Further studies should focus on other metabolites.

Among potentially pathogenic fungal genera, the relative abundance of *Gibberella* significantly increased after adding the upregulated compound mixture compared to the other treatments (Figure 7B), indicating that *Gibberella* responded positively to these substances. In this study, the sugars contained in the upregulated mixture were mannitol and arabinose. Mannitol is not only a photo-assimilate in many plants, some pathogens can also secrete mannitol, which can store or transport carbohydrates for fungi and facilitate fungal spore germination under starvation conditions; thus, it plays an important role in the pathogenicity of fungi (Patel and Williamson, 2016). Whether mannitol can directly promote *Gibberella* growth needs to be further verified. Phloretin is another noteworthy substance in the mixture of upregulated compounds, considered an important autotoxin that causes apple replant disease. *Fusarium* and phloridzin (the glucoside of phloretin) in the soil were significantly positively correlated with apple replant disease, but the direct relationship between phloretin and *Fusarium* has not been clarified (Xiang et al., 2021). The relative abundance of *Gibberella*, *Fusarium*, and *Neocosmospora* significantly decreased after adding the downregulated compound mixture compared to the other treatments (Figure 7B), probably owing to the increase in beneficial bacterial genera *Pseudomonas* and *Bacillus*. *Fusicolla* abundance did not increase after the upregulated mixture treatment but increased after downregulated mixture treatment, this might be due to some substances in the downregulated mixture favoring *Fusicolla* growth; however, the specific relationship between downregulated substances and *Fusicolla* growth has not been reported in the literature, and further verification is needed. The relative abundance of *Mortierella* reduced, and *Trichoderma* did not change after adding a mixture of differentially regulated compounds, suggesting that these two genera are not sensitive to the added substances or do not use these substances as C/N sources. Overall, the changes in some bacterial and fungal genera after exogenously adding the mixtures of differentially regulated compounds verified the “isotope labeling experiment” results. However, since we added a mixture of multiple substances, it was impossible to determine which substance directly affected bacterial or fungal genera, and this needs to be further investigated.

Then, we noticed that 4-HBA treatment inhibited seedlings growth. Our previous study had shown that 4-HBA was quickly adsorbed or transformed after being applied to the soil, and after 48 h of application, the residual content of 4-HBA in the soil was only 0.003% of the added content, and the microorganisms responsible for 4-HBA metabolism in the soil was identified, which may be involved in the N cycle, affecting plant growth (Wang, 2016). Taken together, inhibitory effect of 4-HBA might be attributable to microbial changes rather than the direct effect of 4-HBA. Li et al. (2014a) also reported that the poor performance of the peanut plants was attributed to changes in the soil microbial communities promoted by phenolic acids rather than direct autotoxicity induced by root exudates. In future studies, how these changes in microbial population due to 4-HBA application affect the grapevine plant growth should be further studied.

## Conclusion

In this study, we characterized the effects of root exudates affected by 4-HBA on rhizosphere microorganisms using a combination of metabolomics, DNA-SIP, and high-throughput sequencing. The bacterial and fungal communities of CK-^13^C differed from those of T-^13^C indicating that the 4-HBA treatment changed the composition of the microbial community that assimilate fresh plant root exudates. Specifically, 4-HBA treatment decreased potentially beneficial bacterial genera (*Gemmatimonas*, *Streptomyces*, and *Bacillus*) and increased potentially pathogenic fungi (*Fusarium*, *Neocosmospora*, *Gibberella*, and *Fusicolla*) in ^13^C-labeled microbial communities. Moreover, plant exudation patterns were altered, and most of the amino acids and secondary metabolites were increased, while most of the organic acids, especially fatty acids, were decreased after 4-HBA treatment. The application of a mixture of upregulated compounds to the soil resulted in a decrease in the relative abundance of *Bacillus* (potentially beneficial bacterial genera) and an increase in the relative abundance of *Gibberella* (potentially pathogenic fungi) in the soil compared to the addition of a mixture of downregulated compounds.

**FIGURE 8:**
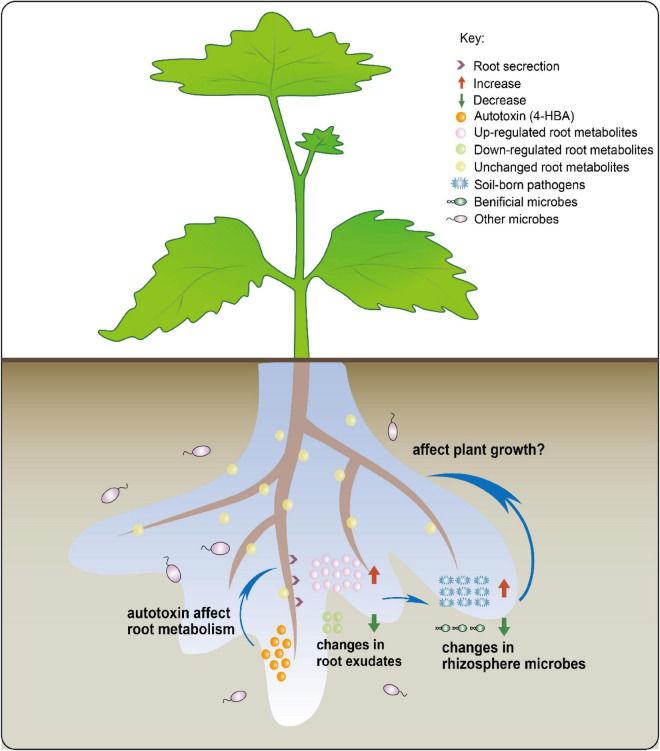
The overview of the mechanism by which autotoxin affects root exudates to change rhizosphere microbiota.

Our combined results allow us to propose a new possible model of rhizosphere response to autotoxins (Figure 8). After continuous cultivation of grapevines, the autotoxins secreted by roots may regulate the rhizosphere microbial community by influencing root secretions and the altered root exudates affecting the rhizosphere microorganisms. However, the regulatory mechanisms of root secretory properties by autotoxins and the effect of microorganisms recruited by root exudates on grapevine seedling growth require further investigation.

## Data availability statement

The datasets presented in this study can be found in online repositories. The names of the repository/repositories and accession number(s) can be found in the article/Supplementary Material.

## Author contributions

KL and XG designed the research. QL performed experiments and wrote the manuscript. LZ, LW, and QW contributed to the interpretation of the data. All authors contributed to the article and approved the submitted version.
