# Supplementary material for: Autotoxin affects the rhizosphere microbial community structure by influencing the secretory characteristics of grapevine roots
Source: Front Microbiol. 2022 Jul 26;13:953424. doi: 10.3389/fmicb.2022.953424 (PMC9360756; doi:10.3389/fmicb.2022.953424)
Supplement: Supplementary file 1 [file Data_Sheet_1.pdf]

Supplementary Material

1 Supplementary Materials and Methods

$^{13}CO_2$-labelling and sampling

$^{13}CO_2$ pulse-labelling was carried out in two climate chambers and labeled with $^{13}CO_2$ from 8 a.m. to 2 p.m. (6h) for seven consecutive days (Fig.1). 15 pots of 4-HBA treated seedlings (T-$^{13}CO_2$) and 15 pots of untreated seedlings (CK-$^{13}CO_2$) were transferred into one chamber, and 5 beakers each containing 10 g of $Na_2{}^{13}CO_2$ (Sigma-Aldrich, MO, USA) were placed in the chamber. Before labelling, the air in the chamber was pump out and returned after through the saturated sodium carbonate solution until the CO_2 concentration in the chamber dropped to about 100 mg·kg^{-1}. The purpose was to consume the original CO_2 in the chamber to promote the subsequent absorption of $^{13}CO_2$. Then, dilute hydrochloric acid was poured into one of the beakers until the $Na_2{}^{13}CO_2$ is completely dissolved to release $^{13}CO_2$ gas. Repeat once every 1 hour until the $Na_2{}^{13}CO_2$ in the 5 beakers were fully dissolved. During the labelling period, the total CO_2 concentration was maintained at 400-500 mg·kg^{-1}. As control, the same number of seedlings (T-$^{12}CO_2$/ CK-$^{12}CO_2$) were placed on the other chamber except for using $Na_2{}^{12}CO_2$ rather than $Na_2{}^{13}CO_2$. Three days after labelling, rhizosphere soils were separately sampled from 15 seedlings of 4 treatments (CK-$^{13}CO_2$/T-$^{13}CO_2$/CK-$^{12}CO_2$/T-$^{12}CO_2$), this experiment was performed on three biological replicates, with five seedlings per biological replicate; and plants after collecting rhizosphere soil are used to collect root exudates, this experiment was performed on six biological replicates, with five seedlings per biological replicate. ^{13}C abundance of rhizosphere soil was determined by isotope ratio mass spectrometry (Isoprime100, IRMS, UK) coupled to an elemental analyzer (Elementar vario PYRO cube， EA, Germany).

Quantitative real-time PCR

Quantitative real-time PCR was used to determine abundance of 16S rRNA gene copy numbers in each fraction with the 338F and 518R primers. The PCR reactions were performed in a 20-μL mixture containing 10 μL of SYBR green PCR Premix Ex Taq II (Takala, Japan), conducted using an ABI Real-Time 7500 system (Applied Biosystems, Waltham, MA, USA) with the following program: 95 °C for 1 min followed by 40 cycles of 94 °C for 15 s, 55 °C for 34 s and 72 °C for 15 s. For measuring 16S rRNA gene quantity, a clone with the correct insert was ligated into the *pEASY*®-T1 vector and transformed into *Trans*1-T1 competent cells. The plasmid was obtained from the clone using a

Miniprep kit (Qiagen, Germantown, MD, USA). Standard curves were obtained by producing a 10-fold serial dilution of plasmid (10^2-10^8 copies), and the R^2 of the standard curve was > 0.99. The qPCRs were run in triplicate using DNA extracted from each soil sample. A blank was always run with water as a template instead of soil DNA extract. To minimize bias, the 16S rRNA gene copies of standard curves and each soil sample were measured at the same time in the same real-time system.

Microbial diversity analysis

The hypervariable V4-V5 region of bacterial 16S rRNA gene fragments was amplified using the 515F/907R primer set. PCR was performed in a final volume of 20 μL containing 4 μL 5× *TransStart* FastPfu Buffer, 2 μL dNTPs (2.5 mM), 0.8 μL of forward (5 μM) and reverse (5 μM) primers, 0.4 μL *TransStart* FastPfu Polymerase, 0.2 μL BSA and 10 ng of template DNA. For fungi community analysis, partial ITS amplicons were produced using the primer set ITS1F/ITS2R. The volumes of PCR reactions were 20 μL with 2 μL of 10× Buffer, 2 μL dNTPs (2.5 mM), 0.8 μL of forward (5 μM) and (5 μM) reverse primers, 0.2 μL rTaq Polymerase (Takala), 0.2 μL BSA and 10 ng of template DNA. PCR was conducted using an ABI GeneAmp® 9700 (Applied Biosystems, Waltham, MA, USA) with the following program: 95 °C for 3 min followed by 30 cycles of 95 °C for 30 s, 55 °C for 30 s and 72 °C for 45 s, and final extension at 72 °C for 10 min. Amplicons were gel purified using the AxyPrepDNA Gel Extraction Kit (Axygen Biosciences, Union City, CA, USA). Purified amplicons were pooled in equimolar amounts and subjected to paired-end sequencing on an Illumina MiSeq PE300 platform (Illumina, San Diego, CA, USA) according to standard protocols of Majorbio Bio-Pharm Technology Co., Ltd. (Shanghai, China). Raw reads were deposited into the NCBI SRA database under accession number PRJAN826998.

The raw reads were demultiplexed, quality-filtered by fastp version 0.20.0 (Chen et al., 2018) and merged by FLASH version 1.2.7 (Magoc and Salzberg, 2011) with the following criteria: (i) the 300 bp reads were truncated at any site receiving an average quality score of <20 over a 50 bp sliding window, and the truncated reads shorter than 50 bp were discarded, reads containing ambiguous characters were also discarded; (ii) only overlapping sequences longer than 10 bp were assembled according to their overlapped sequence. The maximum mismatch ratio of overlap region is 0.2. Reads that could not be assembled were discarded; (iii) Samples were distinguished according to the barcode and primers, and the sequence direction was adjusted, exact barcode matching, 2 nucleotide mismatch in primer matching.

Operational taxonomic units (OTUs) with 97% similarity cutoff were clustered using UPARSE version 7.1(Edgar, 2013), and chimeric sequences were identified and removed. The taxonomy of each OTU representative sequence was analyzed by RDP Classifier version 2.2 against the 16S rRNA database (Release138 http://www.arb-silva.de) and ITS database (Release 8.0 http://unite.ut.ee/index.php) using confidence threshold of 0.7. A subsampling procedure was applied to normalize the number of reads to the minimum observed reads across all samples. Alpha diversity metrics were calculated using Mothur (version v.1.30.2, https://mothur.org/wiki/calculators/) based on normalized data, whereas beta diversity measures and other analyses were calculated using QIIME (Caporaso et al., 2010) and R packages (version 3.3.1) based on normalized data.

Root exudate collection and UHPLC-MS analysis

The plant roots were washed with distilled water and then transferred to a container wrapped in aluminum foil containing 1 L of distilled water to collect the root exudates. During the period, the water was aerated for 15 min at 45-min intervals using an air pump. After 72 h of collection, the collected root exudates samples were stored at -80 °C before further analysis.

Extracts were freeze-dried and were dissolved with 100 μL of acetonitrile-ultrapure water solution (1:1, v/v), centrifuged at 14000g at 4°C for 15 min, and the supernatant were analyzed using an ultra-high performance liquid chromatography (UHPLC) -mass spectrometer MS/ MS system, consisting of Agilent 1290 Infinity LC UHPLC (Agilent Technologies, Inc., Santa Clara, CA) coupled to a Triple TOF 5600+ High Resolution Mass Spectrometer (AB Sciex, Framingham, MA, USA). The UHPLC separation was carried out on a Waters ACQUITY UPLC BEH Amide column (1.7μm, 2.1 mm× 100 mm). The elution was performed by a mixture of eluent A containing 25 μM ammonium and 25 μM acetate ammonia and ultrapure water, as well as eluent B containing acetonitrile, at a flow of 0.3 $mL \cdot mL^{-1}$, with the following gradient elution: 0–0.5 min, 95% eluent B; 0.5–7 min, 95%–65% eluent B; 7–9 min, 65%–40% eluent B; 9–10 min, 40% eluent B; 10–11.1 min, 40%–95% eluent B and 11.1–16 min, 95% eluent B. An MS analysis was used in positive and negative mode using an electrospray ionization (ESI) source. The ESI source conditions were as follows: Ion Source Gas1 (Gas1): 60, Ion Source Gas2 (Gas2): 60, Curtain gas (CUR): 30, source temperature: 600°C, IonSapary Voltage Floating (ISVF) ±5500 V (positive and negative modes); TOF MS scan m/z range: 60-1200 Da, product ion scan m/z range: 25-1200 Da, TOF MS scan accumulation time 0.15 s/spectra, product ion scan accumulation time 0.03 s/spectra ; The secondary mass spectrum was obtained by information dependent acquisition (IDA), and adopts high sensitivity mode, Declustering potential (DP): ±60 V

(both positive and negative modes), Collision Energy: 30 eV, IDA setting was as follows Exclude isotopes within 4 Da , Candidate ions to monitor per cycle: 6. The original data was converted into mzXML format by ProteoWizard, and then the XCMS program was used for peak alignment, retention time correction and peak area extraction.

UHPLC-MS/MS raw data was subjected to a nontargeted metabolomic analysis. The raw data were imported into XCMS software to align the peaks, correct the retention time and extract the peak area. The metabolite structure identification uses accurate mass matching (<25 ppm) and secondary spectrum matching methods to search the laboratory's self-built database. For the data extracted by XCMS, delete ion peaks with missing values >50% in the group, integrate positive and negative ion peaks and apply the software SIMCA-P 14.1 (Umetrics, Umea, Sweden) for pattern recognition. After the data was preprocessed by Pareto-scaling, the results were analyzed in more detail using multi-dimensional analysis orthogonal to partial least squares discriminant analysis (OPLS-DA), principal component analysis (PCA) and single-dimensional analysis (*t*'-test) to screen different metabolites between groups (VIP>1, $p<0.05$). The tools of the KEGG Pathway Database (http://www.genome.jp/kegg/pathway.html) were used to analyze the metabolic pathway.

Reference

Caporaso, J.G., Kuczynski, J., Stombaugh, J., Bittinger, K., Bushman, F.D., Costello, E.K., Fierer, N., Peña, A.G., Goodrich, J.K., Gordon, J.I., Huttley, G.A., Kelley, S.T., Knights, D., Koenig, J.E., Ley, R.E., Lozupone, C.A., McDonald, D., Muegge, B.D., Pirrung, M., Reeder, J., Sevinsky, J.R., Turnbaugh, P.J., Walters, W.A., Widmann, J., Yatsunenko, T., Zaneveld, J., Knight, R., 2010. QIIME allows analysis of high-throughput community sequencing data. Nature Methods 7, 335–336. doi:10.1038/nmeth.f.303

Chen, S., Zhou, Y., Chen, Y., Gu, J., 2018. fastp: an ultra-fast all-in-one FASTQ preprocessor. Bioinformatics 34, i884–i890. doi:10.1093/bioinformatics/bty560

Edgar, R.C., 2013. UPARSE: highly accurate OTU sequences from microbial amplicon reads. Nature Methods 10, 996–998. doi:10.1038/nmeth.2604

Magoc, T., Salzberg, S.L., 2011. FLASH: fast length adjustment of short reads to improve genome assemblies. Bioinformatics 27, 2957–2963. doi:10.1093/bioinformatics/btr507

2 Supplementary Figures

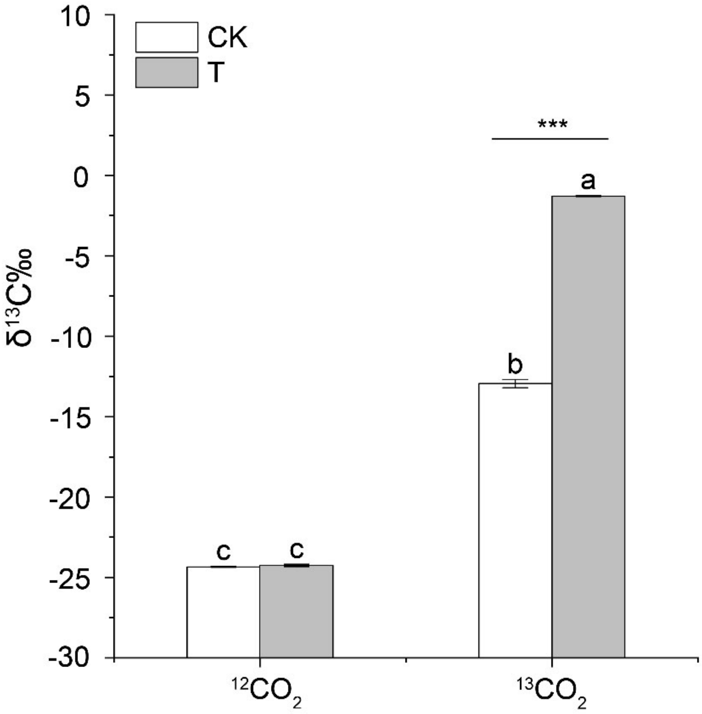

Supplementary Figure S1. The ^{13}C enrichments in the rhizosphere soil of $^{13}CO_2$- or $^{12}CO_2$-labeling. CK: control, T: 4-HBA treatment. Different letters indicated significant differences among the four treatments at the $p < 0.05$ (one-way ANOVA, Trukey's test). Bars represent means ± standard errors (n = 3). *** indicates significant differences between CK-^{13}C and T-^{13}C (Student's *t*-test, $p < 0.001$).

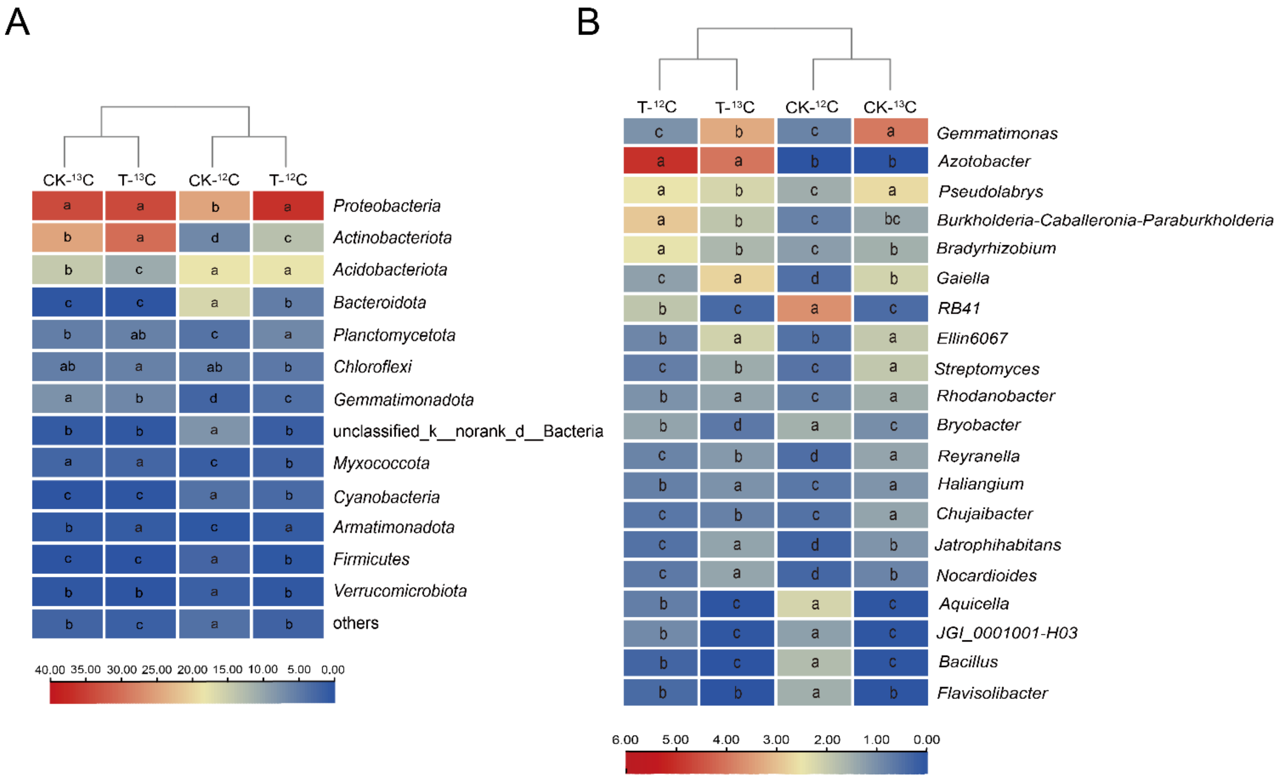

Supplementary Figure S2. A Phyla-level bacterial distribution clustering heatmap in soil samples of ^{12}C- and ^{13}C-labeled DNA under CK and 4-HBA treatments (T). **B** Genus-level bacterial distribution clustering heatmap in soil samples of ^{12}C- and ^{13}C-labeled DNA under CK and 4-HBA treatments (T). Phyla representing less than 1% of the total reads are grouped as “Others.” Different letters indicate significant differences among the four treatments at $p < 0.05$ (one-way ANOVA, Tukey’s test).

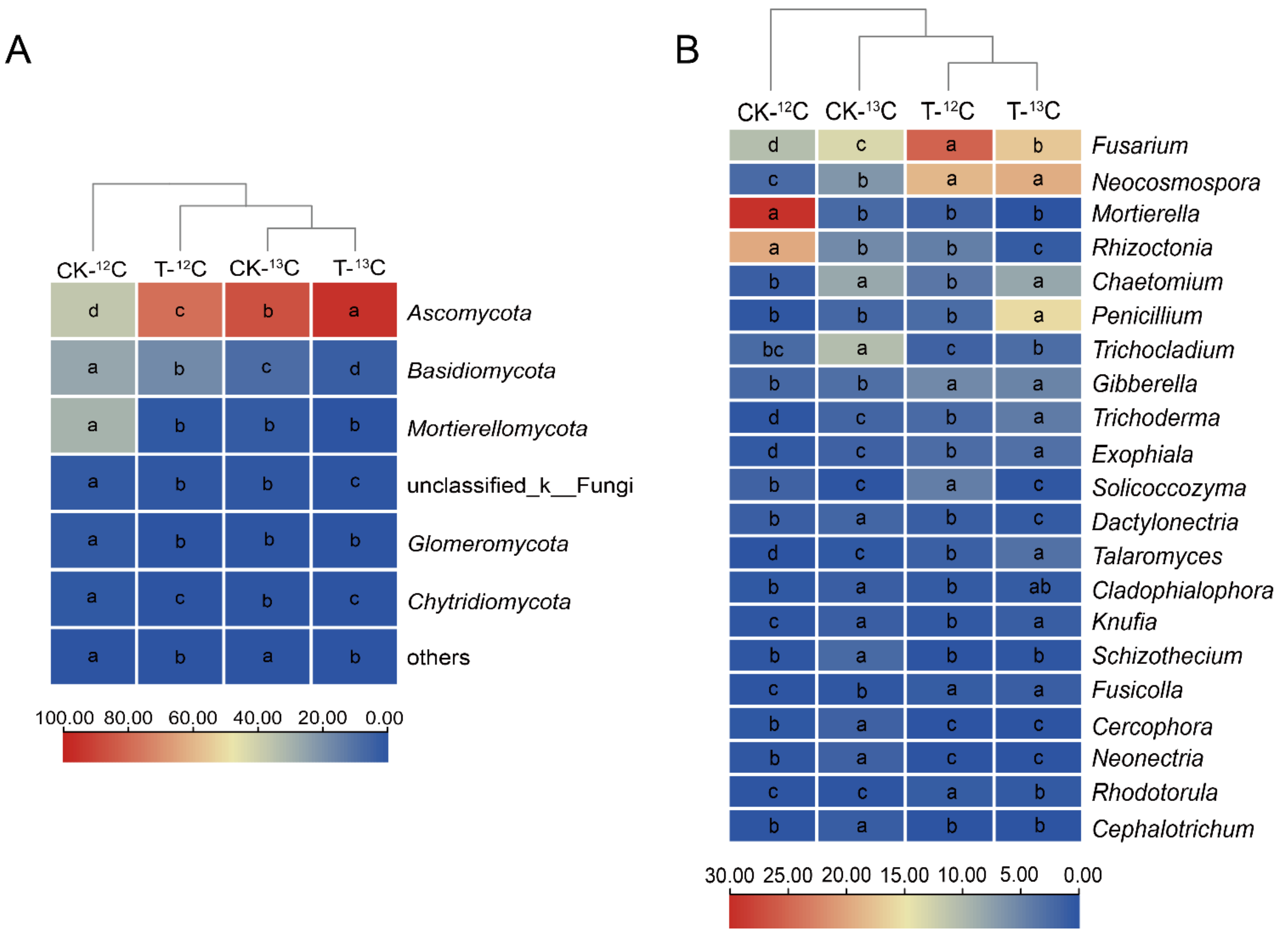

Supplementary Figure S3. A Phyla-level fungal distribution clustering heatmap in soil samples of ^{12}C- and ^{13}C-labeled DNA under CK and 4-HBA treatments (T). **B** Genus-level fungal distribution clustering heatmap in soil samples of ^{12}C- and ^{13}C-labeled DNA under CK and 4-HBA treatments (T). Phyla representing less than 1% of the total reads are grouped as "Others." Different letters indicate significant differences among the four treatments at $p < 0.05$ (one-way ANOVA, Tukey's test).

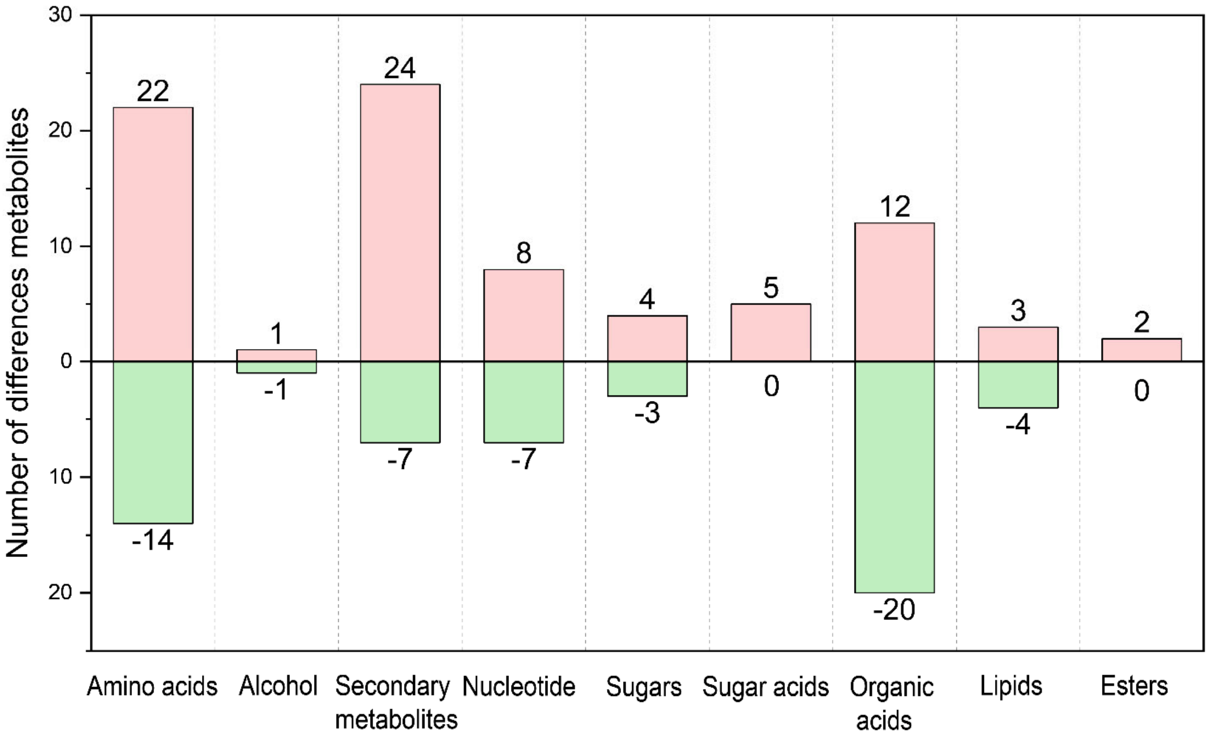

Supplementary Figure S4. The number of upregulated and downregulated metabolites across categories.

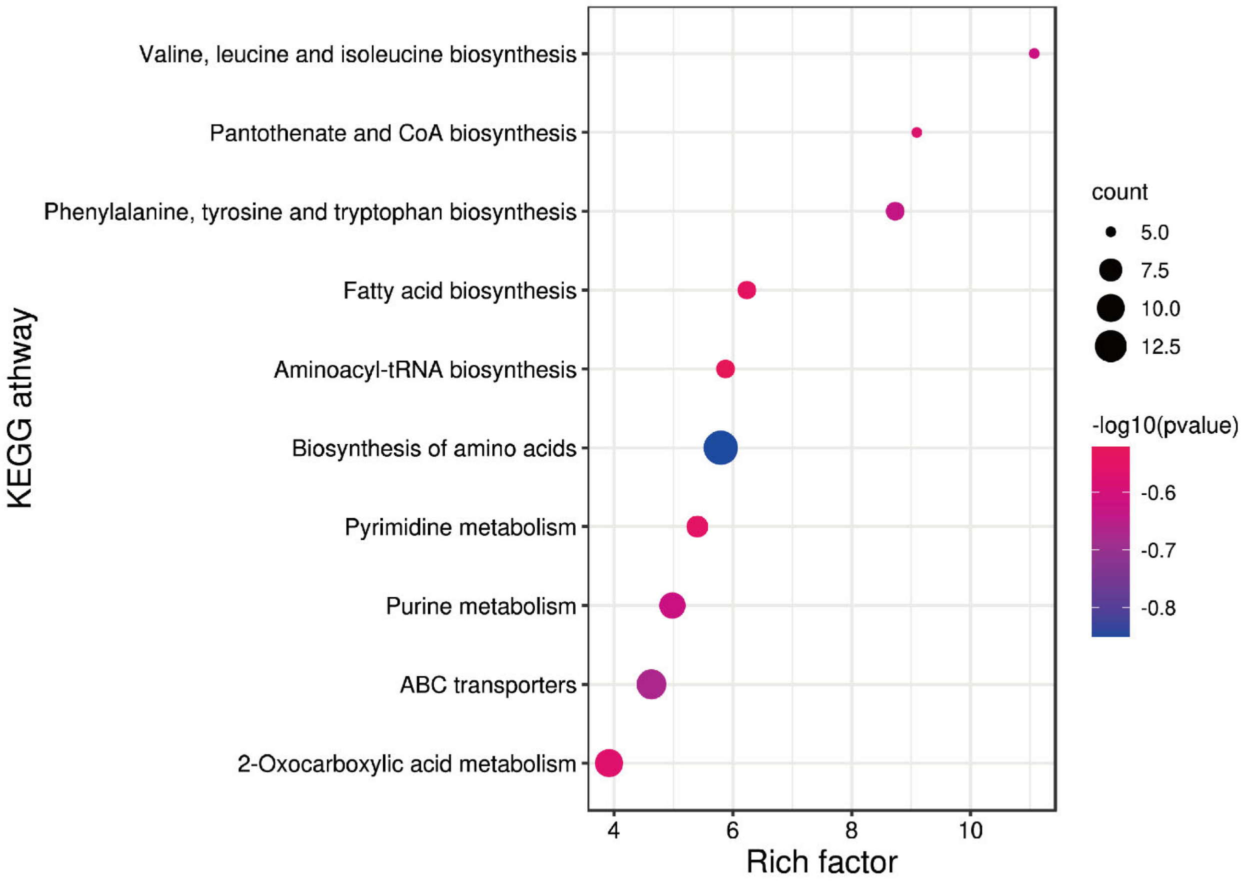

Supplementary Figure S5. Enrichment analysis of the top 10 KEGG metabolic pathways of significantly differential metabolites between CK and 4-HBA treatment.

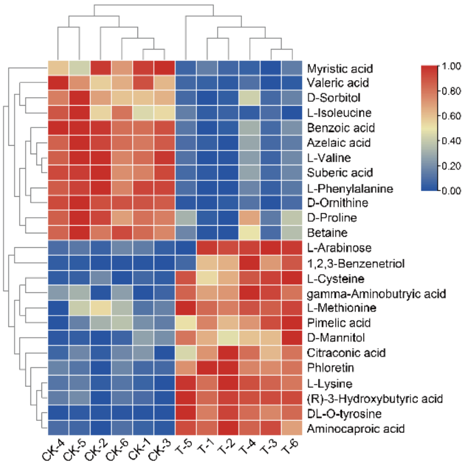

Supplementary Figure S6. Heatmap analysis of changes in abundance of representative compounds significantly different in the root exudates of CK and 4-HBA treatment and selected for the subsequent soil pour back experiment.

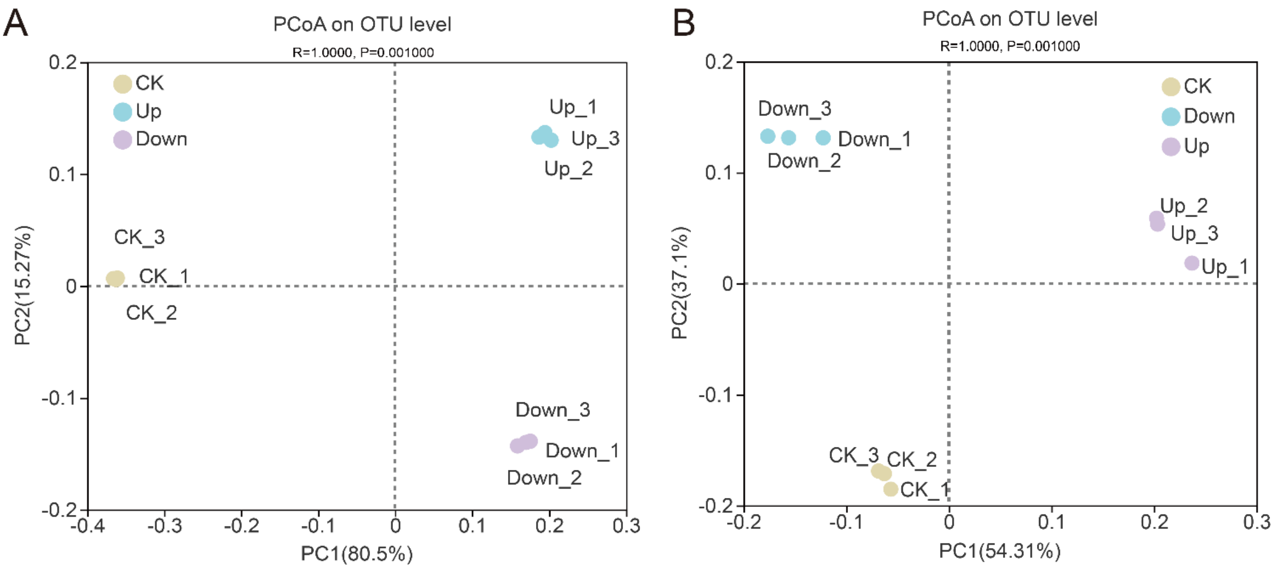

Supplementary Figure S7. **A** Principal co-ordinates analysis based on Bray–Curtis distances of bacterial communities in soil samples after adding upregulated or downregulated compound mixtures. **B** Principal co-ordinates analysis based on Bray–Curtis distances of fungal communities in soil samples after adding upregulated or downregulated compound mixtures.

3 Supplementary Tables

Table S1 Primers used in this study

Target	Primer	Sequence (5'-3')
16S rRNA qPCR	338F	ACTCCTACGGGAGGCAGCAG
	518R	ATTACCGCGGCTGCTGG
16S rRNA amplicon sequencing	515F	GTGCCAGCMGCCGCGG
	907R	CCGTCAATTCMTTTRAGTTT
ITS amplicon sequencing	ITS1F	CTTGGTCATTTAGAGGAAGTAA
	ITS2R	GCTGCGTTCTTCATCGATGC

Table S2 The compounds and its content in the up- and down-regulated solution

	Category	Compound	Dose
Upregulated solutions	Sugars	D-Mannitol	2 mM
		L-Arabinose	2 mM
	Amino acids	DL-O-tyrosine	2 mM
		L-Cysteine	2 mM
		L-Lysine	2 mM
		L-Methionine	2 mM
		γ-aminobutryic acid	2 mM
	Organic acids	Aminocaproic acid	2 mM
		3-Hydroxybutyric acid	2 mM
		Pimelic acid	2 mM
		Citraconic acid	2 mM
	Secondary metabolites	1,2,3-Benzenetriol	2 mM
		Phloretin	2 μM
Downregulated solutions	Sugars	D-Sorbitol	2 mM
	Amino acids	L-Phenylalanine	2 mM
		L-Valine	2 mM
		L-Isoleucine	2 mM
		D-Proline	2 mM
		D-Ornithine	2 mM
	Organic acids	Myristic acid	2 μM
		Azelaic acid	2 mM
		Suberic acid	2 mM
		Valeric acid	2 mM
	Secondary metabolites	Betaine	2 mM
		Benzoic acid	2 mM

Table S3 Relative abundance of bacterial phyla of ^{12}C and ^{13}C-labeled fraction under CK and 4-HBA treatments

Bacterial Phyla	CK-^{12}C (%)	CK-^{13}C (%)	T-^{12}C (%)	T-^{13}C (%)
Proteobacteria	24.94±0.43 b	33.98±0.46 a ***	36.76±0.85 a	34.02±0.78 a
Actinobacteriota	6.70±0.10 d	24.90±0.58 b ***	13.39±0.34 c	30.38±1.13 a ***
Acidobacteriota	18.34±0.50 a **	14.55±0.18 b	18.30±0.29 a ***	10.73±0.25 c
Bacteroidota	16.33±1.19 a ***	0.38±0.03 c	5.31±0.33 b ***	0.25±0.01 c
Planctomycetota	4.09±0.12 c	5.54±0.05 b ***	6.68±0.42 a	5.81±0.23 ab
Chloroflexi	5.29±0.24 ab	5.58±0.11 ab	4.82±0.04 b	5.82±0.23 a *
Gemmatimonadota	2.28±0.05 d	7.74±0.05 a ***	3.71±0.07 c	6.54±0.10 b ***
unclassified_k__norank_d__Bacteria	8.10±1.76 a	1.05±0.08 b	1.51±0.10 b ***	0.65±0.01 b
Myxococcota	1.39±0.06 c	2.38±0.04 a ****	1.98±0.08 b	2.47±0.06 a **
Cyanobacteria	3.97±0.04 a ***	0.20±0.01 c	3.08±0.11 b ***	0.35±0.01 c
Armatimonadota	0.59±0.01 c	0.89±0.02 b ***	1.15±0.07 a	1.08±0.03 a
Firmicutes	2.45±0.09 a ***	0.09±0.02 c	0.58±0.03 b ***	0.06±0.01 c
Verrucomicrobiota	1.65±0.16 a **	0.50±0.03 b	0.67±0.16 b	0.21±0.03 b
Others	3.90±0.10 a ***	2.21±0.06 b	2.07±0.02 b *	1.62±0.11 c

Table S4 Relative abundance of bacterial genus of ^{13}C-labeled fraction under CK and 4-HBA treatments

Bacterial genus	CK-^{12}C (%)	CK-^{13}C (%)	T-^{12}C (%)	T-^{13}C (%)
Gemmatimonas	0.83±0.02 c	3.97±0.03 a ***	1.06±0.05 c	3.31±0.09 b ***
Azotobacter	6.46E-03±2.44E-03 b	4.16E-03±1.39E-03 b	5.00±0.16 a	4.02±0.38 a
Pseudolabrys	1.51±0.04 c	2.65±0.07 a ***	2.48±0.06 a *	2.20±0.02 b
Burkholderia_Caballeronia_Paraburkholderia	0.79±0.12 c	1.42±0.08 bc *	2.91±0.28 a *	1.91±0.07 b
Bradyrhizobium	1.25± 0.01 c	1.53±0.03 b ***	2.45±0.06 a ***	1.65±0.04 b
Gaiella	0.51±0.01 d	2.15±0.06 b ***	1.29±0.08 c	2.75±0.14 a ***
RB41	3.66±0.40 a ***	0.44±0.03 c	1.91±0.21 b **	0.39±0.01 c
Ellin6067	0.54±0.07 b	1.96±0.03 a ***	0.87±0.07 b	2.14±0.16 a **
Streptomyces	0.57±0.06 c	1.94±0.02 a ***	0.76±0.03 c	1.47±0.07 b ***
Rhodanobacter	0.78±0.05 c	1.51±0.06 a ***	1.05±0.05 b	1.35±0.07 a*
Bryobacter	1.54±0.03 a ***	0.99±0.03 c	1.35±0.03 b ***	0.65±0.02 d
Reyranella	0.48±0.04 d	1.36±0.02 a ***	0.83±0.04 c	1.16±0.04 b **
Haliangium	0.63±0.01 c	1.09±0.01 a ***	0.76±0.04 b	1.06±0.03 a **
Chujaibacter	0.54±0.02 c	1.35±0.04 a ***	0.63±0.03 c	0.78±0.01 b *
Jatrophihabitans	0.28±0.01 d	1.10±0.03 b ***	0.53±0.02 c	1.34±0.07 a ***
Nocardioides	0.34±0.01 d	0.83±0.02 b ***	0.66±0.04 c	1.36±0.05 a ***
Aquicella	2.19±3.33E-03 a ***	0.05±3.33E-03 c	0.74±0.03 b ***	0.03±6.05E-03 c
JGI_0001001_H03	1.29±0.07 a **	0.04±5.62E-03 c	0.94±0.14 b *	0.05±4.02E-03 c
Bacillus	1.74±0.06 a ***	0.04±4.23E-03 c	0.30±0.01 b**	0.01±9.23E-04 c
Flavisolibacter	1.52±0.18 a *	0.03±9.77E-03 b	0.42±0.04 b **	0.01±9.23E-04 b

Table S5 Comparison on relative abundance of bacterial phyla between CK-^{13}C and T-^{13}C

Bacterial Phyla	CK-^{13}C (%)	T-^{13}C (%)	logFC	*p*-value
Proteobacteria	33.98±0.46	34.02±0.78	0.0005	9.62E-01
Actinobacteriota	24.90±0.58	30.38±1.13	0.09	1.26E-02
Acidobacteriota	14.55±0.18	10.73±0.25	-0.13	2.51E-04
Bacteroidota	0.38±0.03	0.25±0.01	-0.19	9.70E-03
Planctomycetota	5.54±0.05	5.81±0.23	0.02	3.06E-01
Chloroflexi	5.58±0.11	5.82±0.23	0.02	4.06E-01
Gemmatimonadota	7.74±0.05	6.54±0.10	-0.07	4.84E-04
Myxococcota	2.38±0.04	2.47±0.06	0.01	3.11E-01
Cyanobacteria	0.20±0.01	0.35±0.01	0.23	7.76E-04
Armatimonadota	0.89±0.02	1.08±0.03	0.08	4.42E-03
Firmicutes	0.09±0.02	0.06±0.01	-0.12	3.44E-01
Verrucomicrobiota	0.50±0.03	0.21±0.03	-0.38	2.94E-03

Table S6 Comparison on relative abundance of bacterial genus between CK-^{13}C and T-^{13}C

Bacterial genus	CK-^{13}C (%)	T-^{13}C (%)	logFC	*p*-value
Gemmatimonas	3.97±0.03	3.31±0.09	-0.08	2.40E-03
Azotobacter	4.16E-03±1.39E-03	4.02±0.38	3.16	4.77E-04
Pseudolabrys	2.65±0.07	2.20±0.02	-0.08	3.11E-03
Burkholderia_Caballeronia_Paraburkholderia	1.42±0.08	1.91±0.07	0.13	1.04E-02
Bradyrhizobium	1.53±0.03	1.65±0.04	0.03	8.63E-02
Gaiella	2.15±0.06	2.75±0.14	0.11	1.78E-02
RB41	0.44±0.03	0.39±0.01	-0.05	2.05E-01
Ellin6067	1.96±0.03	2.14±0.16	0.04	3.16E-01
Streptomyces	1.94±0.02	1.47±0.07	-0.12	2.99E-03
Rhodanobacter	1.51±0.06	1.35±0.07	-0.05	1.41E-01
Bryobacter	0.99±0.03	0.65±0.02	-0.18	6.90E-04
Reyranella	1.36±0.02	1.16±0.04	-0.07	1.19E-02
Haliangium	1.09±0.01	1.06±0.03	-0.01	4.65E-01
Chujaibacter	1.35±0.04	0.78±0.01	-0.24	2.00E-04
Jatrophihabitans	1.10±0.03	1.34±0.07	0.09	3.24E-02
Nocardioides	0.83±0.02	1.36±0.05	0.21	7.51E-04
Aquicella	0.05±3.33E-03	0.03±6.05E-03	-0.29	2.26E-02
JGI_0001001_H03	0.04±5.62E-03	0.05±4.02E-03	-0.51	7.68E-02
Bacillus	0.04±4.23E-03	0.01±9.23E-04	-0.42	3.48E-03
Flavisolibacter	0.03±9.77E-03	0.01±9.23E-04	-0.02	1.19E-01

Table S7 Relative abundance of fungal phyla of ^{12}C and ^{13}C-labeled fraction under CK and 4-HBA treatments

Fungal Phyla	CK-^{12}C	CK-^{13}C	T-^{12}C	T-^{13}C
Ascomycota	36.979±0.789 d	86.93±0.71 b ***	78.83±0.55 c	95.97±0.68 a ***
Basidiomycota	26.47±0.80 a ***	8.47±0.64 c	18.08±0.48 b ***	3.53±0.63 d
Mortierellomycota	30.26±1.08 a ***	2.44±0.11 b	1.69±0.03 b ***	0.10±0.03 b
unclassified_k__Fungi	2.25±0.09 a ***	1.01±0.12 b	1.07±0.07 b ***	0.34±0.06 c
Glomeromycota	2.21±0.31 a **	0.56±0.02 b	0.15±3.9E-03 b *	0.04±0.02 b
Chytridiomycota	1.71±0.07 a ***	0.52±0.12 b	0.16±0.02 c **	0.02±6.3E-03 c
Others	0.13±0.02 a	0.08±0.01 a	0.02±2.1E-03 b *	5.2E-03±5.2E-04 b

Table S8 Relative abundance of fungal genus of ^{12}C and ^{13}C-labeled fraction under CK and 4-HBA treatments

Fungal genus	CK-^{12}C	CK-^{13}C	T-^{12}C	T-^{13}C
Fusarium	10.57±0.49 d	13.46±0.24 c **	25.44±0.44 a ***	17.35±0.16 b
Neocosmospora	2.40±0.23 c	6.69±0.24 b ***	18.69±1.09 a	19.44±0.26 a
Mortierella	29.75±1.08 a **	2.36±0.12 b	1.66±2.71E-02 b ***	8.63E-02±2.25E-02 b
Rhizoctonia	19.75±0.51 a ***	5.62±0.65 b	4.42±4.85E-02 b ***	0.88±0.18 c
Chaetomium	1.21±4.18E-02 b	8.45±1.09 a *	3.70±0.57 b	8.29±1.02 a *
Penicillium	0.39±5.79E-02 b	2.12±0.48 b *	2.60±0.96 b	15.84±2.28 a **
Trichocladium	2.53±0.10 bc	10.45±0.38 a ***	1.60±0.13 c	2.73±0.22 b *
Gibberella	2.22±0.14 b	2.91±0.22 b	5.47±0.13 a	4.93±0.18 a
Trichoderma	0.25±3.33E-02 d	1.81±8.44E-02 c ***	2.47±0.18 b	4.08±0.11 a **
Exophiala	0.67±4.34E-02 d	1.54±7.23E-02 c ***	2.64±7.35E-02 b	3.02±8.35E-02 a *
Solicoccozyma	1.58±5.90E-02 b ***	0.22±5.87E-02 c	4.37±0.37 a **	0.40±8.07E-02 c
Dactylonectria	1.26±6.49E-02 b	1.93±3.13E-02 a **	1.14±5.79E-02 b **	0.76±4.43E-02 c
Talaromyces	4.52E-02±3.93E-03 d	0.56±0.11 c *	1.27±2.34E-02 b	3.14±0.15 a ***
Cladophialophora	0.56±3.84E-02 b	1.26±0.15 a	0.80±9.92E-03 b	0.93±0.12 ab
Knufia	2.84E-01±1.22E-02 c	1.20±5.49E-02 a ***	5.73E-01±1.56E-02 b	1.12±5.03E-02 a ***
Schizothecium	0.30±1.40E-02 b	2.38±0.11 a **	0.11±9.63E-03 b	0.27±3.25E-02 b **
Fusicolla	0.19±1.13E-02 c	0.41±4.55E-02 b**	1.01±4.10E-02 a	1.21±6.59E-02 a
Cercophora	0.47±4.80E-02 b	1.50±0.12 a **	4.16E-02±2.08E-03 c	8.53E-02±1.98E-02 c
Neonectria	0.36±6.06E-02 b	1.49±2.74E-02 a ***	5.30E-02±8.87E-03 c	0.12±1.04E-02 c **
Rhodotorula	0.16±3.52E-02 c	0.15±3.24E-02 c	1.02±6.90E-02 a	0.68±0.12 b
Cephalotrichum	0.15±9.91E-03 b	1.03±8.01E-02 a **	4.78E-02±1.04E-03 b	0.11±2.69E-02 b

Table S9 Comparison on relative abundance of fungal phyla between CK-^{13}C and T-^{13}C

Fungal Phyla	CK-^{13}C	T-^{13}C	logFC	*p*-value
Ascomycota	86.93±0.71	95.97±0.68	0.04	7.78E-04
Basidiomycota	8.47±0.64	3.53±0.63	-0.38	5.34E-03
Mortierellomycota	2.44±0.11	0.10±0.03	-1.41	3.31E-05
Glomeromycota	0.56±0.02	0.04±0.02	-1.17	4.19E-05
Chytridiomycota	0.52±0.12	0.02±6.3E-03	-1.39	1.59E-02

Table S10 Comparison on relative abundance of fungal genus between CK-^{13}C and T-^{13}C

Fungal genus	CK-^{13}C	T-^{13}C	logFC	*p*-value
Fusarium	13.46±0.24	17.35±0.16	0.11	1.79E-04
Neocosmospora	6.69±0.24	19.44±0.26	0.46	3.59E-06
Mortierella	2.36±0.12	8.63E-02±2.25E-02	-1.44	4.24E-05
Rhizoctonia	5.62±0.65	0.88±0.18	-0.80	2.16E-03
Chaetomium	8.45±1.09	8.29±1.02	-0.01	9.22E-01
Penicillium	2.12±0.48	15.84±2.28	0.87	4.17E-03
Trichocladium	10.45±0.38	2.73±0.22	-0.58	6.02E-05
Gibberella	2.91±0.22	4.93±0.18	0.23	1.98E-03
Trichoderma	1.81±8.44E-02	4.08±0.11	0.35	7.58E-05
Exophiala	1.54±7.23E-02	3.02±8.35E-02	0.29	1.79E-04
Solicoccozyma	0.22±5.87E-02	0.40±8.07E-02	0.25	1.59E-01
Dactylonectria	1.93±3.13E-02	0.76±4.43E-02	-0.41	2.69E-05
Talaromyces	0.56±0.11	3.14±0.15	0.75	1.41E-04
Cladophialophora	1.26±0.15	0.93±0.12	-0.13	1.60E-01
Knufia	1.20±5.49E-02	1.12±5.03E-02	-0.03	3.51E-01
Schizothecium	2.38±0.11	0.27±3.25E-02	-0.95	4.34E-05
Fusicolla	0.41±4.55E-02	1.21±6.59E-02	0.46	5.89E-04
Cercophora	1.50±0.12	8.53E-02±1.98E-02	-1.25	3.22E-04
Neonectria	1.49±2.74E-02	0.12±1.04E-02	-1.09	1.24E-06
Rhodotorula	0.15±3.24E-02	0.68±0.12	0.64	1.24E-02
Cephalotrichum	1.03±8.01E-02	0.11±2.69E-02	-0.98	3.95E-04

Table S11 Metabolites detected from grapevine root secretions by UHPLC-MS. Those marked in yellow are the significantly different metabolites. The data were shown in the **Dataset (Excel sheet)**
